# Cyto-Genotoxic Impacts of Antimony Tin Oxide (ATO) Nanoparticles on *Allium cepa* Root Meristem Cells: An Integrative Experimental and in Silico Approach

**DOI:** 10.1021/acsomega.5c08687

**Published:** 2026-01-23

**Authors:** Recep Liman, Erman Salih Istifli, Yaser Acikbas, Yudum Yeltekin Uğur, Maria Suciu, Lucian Barbu-Tudoran, I.˙brahim Hakkı Ciğerci

**Affiliations:** † Molecular Biology and Genetics Department, Faculty of Engineering and Natural Sciences, 175652 Uşak University, Usak 64300, Turkiye; ‡ Biology Department, Faculty of Science and Literature, Cukurova University, Adana 01330, Turkiye; § Electrical and Electronic Engineering Department, Faculty of Engineering and Natural Sciences, Uşak University, Usak 64300, Turkiye; ∥ 61787 National Institute for Research and Development of Isotopic and Molecular Technologies (INCDTIM), Cluj-Napoca 400293, Romania; ⊥ Electron Microscopy Center “C. Craciun”, Faculty of Biology and Geology, Babes-Bolyai University, Cluj-Napoca 400006, Romania; # Molecular Biology and Genetics Department, Faculty of Science and Literatures, 53002 Afyon Kocatepe University, Afyon 03200, Turkiye

## Abstract

In this study, antimony tin oxide (ATO) nanoparticles (NPs) were evaluated for their cyto-genotoxic effects on *Allium cepa* root tips, as their widespread use in industrial and electronic applications raises concerns about possible environmental release and biological hazards that remain largely unexplored. The ATO NPs were characterized using high-resolution transmission electron microscopy (HRTEM), energy-dispersive X-ray spectroscopy (EDX), and X-ray diffraction (XRD), confirming high structural uniformity and crystallinity. Root tips were exposed to 12.5, 25, 50, and 100 μg/mL of ATO NPs for 4 h, and cyto-genotoxicity was assessed using the Allium anaphase-telophase and alkaline comet assays. ATO NPs caused a significant concentration-dependent decrease in mitotic index (MI) and an increase in chromosomal aberrations (CAs) such as laggards, bridges, stickiness, and polyploidy along with DNA damage, indicating suppressed cell division and genotoxic potential of ATO NPs in plant cells, respectively. SEM and TEM analysis also revealed morphological alterations in treated roots compared to controls. In computational docking, the ATO NP showed more favorable predicted affinities against the colchicine-binding site of the tubulin heterodimer (Δ*G* = −11.06 kcal/mol) and the synthetic B-DNA dodecamer (Δ*G* = −12.07 kcal/mol) than colchicine and methylmethanesulfonate (MMS), respectively, suggesting a possible dual genotoxic pathway involving microtubule perturbation and DNA interaction. Consequently, this study demonstrated that ATO NPs induce cyto- genotoxic effects in *A. cepa* root meristematic cells, supporting the use of this model as a reliable tool for NP toxicity assessment.

## Graphical abstract

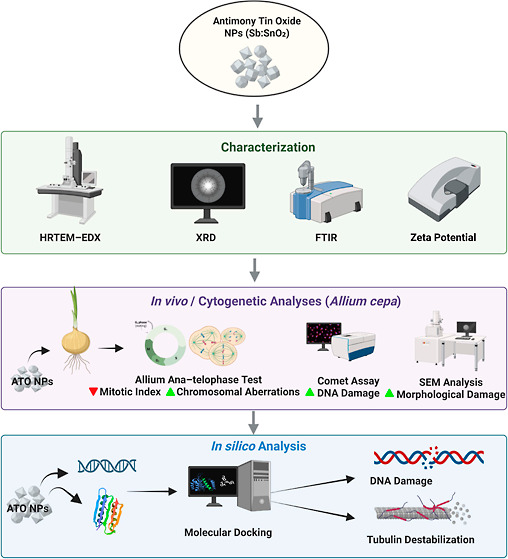

## 1 Introduction

Antimony tin oxide (ATO) nanoparticles (NPs) are typically composed of tin oxide (SnO_2_) doped with antimony (Sb). ATO NPs exhibit important electrical and optical properties, making them highly suitable for applications in optoelectronic and flat-panel display technologies, including light-emitting diodes liquid crystal displays and electrochromic displays.^1,2^ Additionally, they are employed in the development of solar cells^3^ and transparent electrodes^4^ due to their excellent conductivity and transparency. Their high electrical conductivity further enables their use as antistatic agents in industries involving coatings,^5^ chemical fibers,^6^ and polymer membranes.^7^ Moreover, ATO NPs demonstrate significant thermal stability, allowing their incorporation in thermal insulation applications, such as heat-shielding coatings for buildings, glass, and hot mirrors.^8–10^ They are also utilized in automotive and aerospace glazing to prevent fogging and frost formation. Furthermore, ATO NPs possess the ability to attenuate microwave radiation, rendering them effective in electromagnetic interference. This makes them valuable in environments requiring electromagnetic protection, such as radar systems and computer facilities shielding.^11–13^ ATO NPs have been also used as a potential photothermal tool in anticancer therapies due to their wavelength-tunable photothermal conversion, colloidal stability in cell culture mediums, and biocompatibility.^14,15^

Growing industrial use of ATO NPs increases the likelihood of their unintentional release into the environment through manufacturing, usage, and disposal processes. Once released, these NPs can interact with various environmental organisms, including plants, soil invertebrates, and aquatic species. Such interactions may induce oxidative stress, growth inhibition, genotoxicity, and alterations in physiological and biochemical processes. Therefore, understanding the environmental toxicity of ATO NPs is crucial for evaluating their potential ecological risks and ensuring their safe and sustainable application. According to a Park et al. study, ATO NPs at a concentration of 1 mg/mL dramatically decreased the potency of *Staphylococcus aureus* and uropathogenic *Escherichia coli* and prevented them from forming biofilms. This suggests that it might be used as an antibacterial agent.^16^ Since no acute toxicity was observed in zebrafish adults or eggs/embryos, commercial ATO NPs appear to be rather safe for aquatic life. However, the notable changes were observed in heartbeat and spontaneous movements and slight bioaccumulation in mussels that were noticed warrant further research.^17^

The Allium test is one of the most utilized plant-based cytogenetic bioassays for assessing the cyto-genotoxic potential of chemical substances, particularly those posing environmental risks due to their uncontrolled release. This test offers numerous advantages, including a short experimental duration, ease of application, cost efficiency, high reproducibility, and the presence of relatively few but large chromosomes (2n = 16), which facilitates microscopic analysis. Moreover, its outcomes often show strong correlation with results from other biological assays.^18–20^ In recent years, the Allium test has been increasingly employed to evaluate the genotoxic effects of micro- and nanomaterials, serving as a reliable biomarker in environmental genotoxicology.^21–24^ Complementary to this, the comet assay—also known as single-cell gel electrophoresis (SCGE)—is frequently used to detect DNA strand breaks in the root meristematic cells of *Allium cepa*. Its popularity in NP toxicity research stems from its simplicity, sensitivity, adaptability, and cost-effectiveness. The method is particularly effective in revealing DNA damage induced by NPs like ATO, allowing the quantification of genotoxicity at the level of individual cells.^25–28^

Molecular docking, in the past decade, has emerged as a cornerstone bioinformatics tool for deciphering the genotoxic mechanisms of small molecules—including NPs—by quantitatively describing their interactions with intracellular targets.^29–32^ By predicting the most favorable binding orientations and associated energetics (binding affinity), docking provides valuable mechanistic insights that complement experimental observations.^33^ Given that the cyto-genotoxic profile of ATO NPs remain largely unexplored, in silico interrogation of their potential cellular biomacromolecular targets offers a powerful strategy to clarify their mode of action at the molecular level and to provide mechanistic basis for future experimental studies.

In this study, ATO NPs were characterized using HRTEM-EDX, XRD, and Zetasizer to determine their functional groups, crystalline structure, electrostatic dissipation mechanism and morphological properties. The Allium ana-telophase and alkaline comet assays were employed to assess the cyto-genotoxic responses induced by ATO NPs exposure at various concentrations for 4 h. To our knowledge, this study represents one of the first comprehensive evaluations of ATO-induced cyto-genotoxicity in plant-based assay systems. Furthermore, molecular docking simulations were performed to explore the potential binding interactions between ATO NPs and the colchicine-binding pocket of the tubulin heterodimer and the synthetic B-DNA dodecamer providing insight into possible mechanisms underlying ATO NPs-induced cyto-genotoxic damage.

The aim of this study was to elucidate the cyto-genotoxic effects and potential mechanisms of ATO NPs using *A. cepa* as a sensitive plant bioindicator system. Given the extensive use of ATO NPs in optoelectronic devices, coatings, and biomedical applications, their inevitable release into the environment raises concerns about potential ecological and biological risks. Despite their growing industrial importance, data on the cellular and molecular toxicity of ATO NPs remain scarce, particularly in plants that play key roles in ecosystem health. Therefore, this study sought to comprehensively characterize ATO NPs using HRTEM-EDX, XRD, and Zetasizer techniques and to assess their cyto-genotoxic impacts through *Allium* ana-telophase and alkaline comet assays. Furthermore, molecular docking simulations targeting the tubulin heterodimer and DNA were performed to provide mechanistic insight into their interactions at the molecular level. Collectively, this integrative approach aimed to clarify the biological hazards associated with ATO NPs and contribute to the development of safer nanomaterial applications.

## 2 Materials and Methods

### 2.1 Organism

Bulbs of *A. cepa* L. (2n = 16), 25–30 mm in diameter, were procured from a local market and used without any pretreatment.

### 2.2 Chemicals

Antimony tin oxide (ATO) Nanopowder/Nanoparticles (Purity: 99.9%) was supplied from Nanografi (ODTU, Teknopark, Turkiye). All other chemicals were analytical grade and purchased from commercial suppliers.

### 2.3 Characterization of ATO NPs

The morphology and crystallinity of the ATO NPs were analyzed using HRTEM (Jeol 2100F HRTEM) equipped with EDX. Prior to analysis, the powdered sample was suspended in ethyl alcohol and stirred in an ultrasonic cleaner for 60 min. Then, a drop was placed on the grid with a micropipette and left to dry for at least one night. The measurements were taken using a carbon-coated film grid and a CF200–Cu carbon film grid. XRD analyses of the ATO NPs were performed using a Rigaku brand Miniflex model desktop device with a copper (Cu) X-ray tube and Cu–Kα X-ray with a wavelength of 1.544 Å. X-ray diffraction patterns were measured in the 3–90° (2θ) range. The Zeta potential of ATO NPs was also analyzed in water by Zetasizer (Ver. 7.13, Malvern). The XRD patterns of the ATO NPs (see Figure S1), the EDX spectra (see Figure S2) and the corresponding HRTEM micrographs (see inset images in Figure S2) are presented in the Supporting Information, in addition to the zeta potential measurements, the average particle size and the polydispersity index (PDI) of the ATO NP dispersion.

### 2.4 *Allium cepa* Ana-Telophase Test

ATO NPs were suspended in distilled water to obtain final concentrations of 12.5, 25, 50, and 100 μg/mL. To ensure uniform dispersion and minimize agglomeration, the suspensions were treated in a bath sonicator (Bandelin Sonorex Digitec DT100, Germany) at 320 W and 35 kHz for 30 min at room temperature. The freshly prepared suspensions were used immediately for exposure experiments and were not stored for extended periods to prevent particle aggregation or sedimentation. All procedures were performed under clean laboratory conditions to maintain sample integrity and reproducibility. These concentrations and application time fell within the range mentioned in earlier research on exposure of NPs to *A. cepa* root tips.^21,27,34,35^ The ana-telophase test was carried out following the method detailed by Liman et al.^27^ Onion bulbs pregerminated in distilled water at room temperature for 48 h were exposed to ATO NPs treatments for 4 h under dark conditions. For comparison, 10 μg/mL methylmethanesulfonate (MMS) was used as a positive control, while distilled water served as the negative control. Each group consisted of three biological replicates. After exposure, approximately 5–6 root tips (around 1 cm in length) were excised from each bulb and fixed in a 3:1 mixture of ethanol and glacial acetic acid at 4 °C for 24 h. Postfixation, the roots were rinsed with distilled water and stored in 70% ethanol at 4 °C. For cytological analysis, the root tips were again rinsed, hydrolyzed in 1 N HCl at 60 °C for 8–10 min, and stained with Schiff’s reagent for 20–25 min at room temperature. A Nikon Eclipse Ci-L light microscope (Japan) equipped with a CMOS camera (Argenit, Cameram5, Turkey) was used to view the slides made using the root tip squash technique. About 1012–1377 cells were examined for mitotic phases and mitotic index (MI) for each treatment, and 500 anaphase-telophase cells were evaluated for chromosomal aberrations (CAs) in five separate samples from each treatment group. The formulas listed below were used

1 $$MI=\frac{number\;of\;cells\;in\mathrm{div}ision}{number\;of\;total\;cells}\times 100$$

2 $$phaseindex=\frac{particular\;phase}{number\;of\;cells\;in\mathrm{div}ision}\times 100$$

3 $$CA=\frac{total\;ana-telophase\;anomalies}{100ana-telophase\;cells}\times 100$$

### 2.5 Scanning Electron Microscopy (SEM) and Transmission Electron Microscopy (TEM) on Allium Roots

Five onion roots from the untreated and ATO NPs-treated roots were freshly cut and fixed in 2.5% glutaraldehyde in phosphate buffer saline (PBS) for 1.5 h, then washed for 3 × 15 min with PBS and then another 15 min wash in distilled water. The roots were left to dry at air for 24 h and then were mounted on a sample holder using a carbon sticky tape and were sputter-coated with 10 nm Au. Images were obtained on a Hitachi SU8230 scanning electron microscope at 30 kV, 10 μA and 10 mm working distance conditions. The root tip and the absorbant region of the roots were imaged at 30×–1000× magnifications. Electron dispersive X-ray analyses (EDX) were done on the same samples using the Oxford Instruments X-MaxN 80 EDX detector and the associated AZtec 3.3 software.

Five onion roots from the untreated and ATO-treated lots were prepared for TEM according to previously described protocol.^36^ In short, roots tips were cut into 1 mm^3^ pieces and fixed in 2.5% glutaraldehyde in for 1.5 h, then washed for 3 × 15 min with PBS. A second fixing was done in OsO_4_ for another 1.5 h, then washed again for 3 × 15 min with PBS. Samples were further dehydrated in increasing acetone solutions of 30–50–75–90–100%, 15 min per step and then gradually infiltrated with Epon 812 using mixed Epon to acetone solutions (3:1, 1:1, 1:3) for 1 h per step, and then pure Epon 2 × 1 h and an overnight infiltration. Samples were then placed in capsules and left to cure for 3 days at 60 °C. Each sample was then trimmed and sectioned at 70–100 nm thickness slices. Sections were collected on 200 mesh Cu grids and stained for contrast with Uranyless and Pb citrate, washed with distilled water and left to air-dry. Images were taken on Hitachi SU8230 SEM at 30 kV, 10 μA equipped with a transmission detector and EDX point analyses were done on the same samples and areas containing NP-like inclusions to confirm the NPs presence, using the equipment and software previously described.

### 2.6 Comet Assay

A modified comet assay^19^ was used to assess ATO NPs-induced DNA damage under the same treatment conditions as the Allium test. Every treatment was carried out in triplicate under dark conditions. From each bulb, 7–8 root tips (∼0.5 cm) were finely chopped in 600 μL of chilled Tris-MgCl_2_ buffer (0.2 M Tris, 4 mM MgCl_2_·6H_2_O, 0.5% Triton X-100, pH 7.5) using a razor blade. The suspension was filtered through a 50 μm nylon mesh, transferred to Eppendorf tubes, and centrifuged at 2100 rpm at 4 °C to pellet the nuclei. After discarding the supernatant, the nuclei were resuspended in PBS. The preparation of the slides followed the guidelines previously outlined by Yardimci et al.^34^ For slide preparation, 100 μL of the nuclear suspension was mixed with 100 μL 1.5% low melting point agarose, layered onto slides precoated with 1% normal melting point agarose, and immediately covered with coverslips. Slides were electrophoresed at 25 V and 300 mA for 20 min at 4 °C after being kept for 20 min in an alkaline buffer (1 mM EDTA, 300 mM NaOH, pH > 13). Slides were then neutralized for 5 min in 400 mM Tris (pH 7.4), rinsed, and stained with 20 μg/mL ethidium bromide for 5 min. Three slides per treatment were randomly coded and scored blindly. From each slide, 50 nuclei were chosen at random and evaluated according to a five-point damage classification scale (ranging from 0 to 4), based on the structure of the comet tail and the degree of DNA migration, as shown in Figure 1, using a fluorescence microscope equipped with a CCD camera (BAB, TAM-F and TC-5, Turkey). The following formula could be used to determine the total amount of DNA damage (eq 4).

4 $$arbitrary\;unit\;\left(AU\right)=\sum_{i=0}^{4}Ni\times i$$

**Figure 1:**
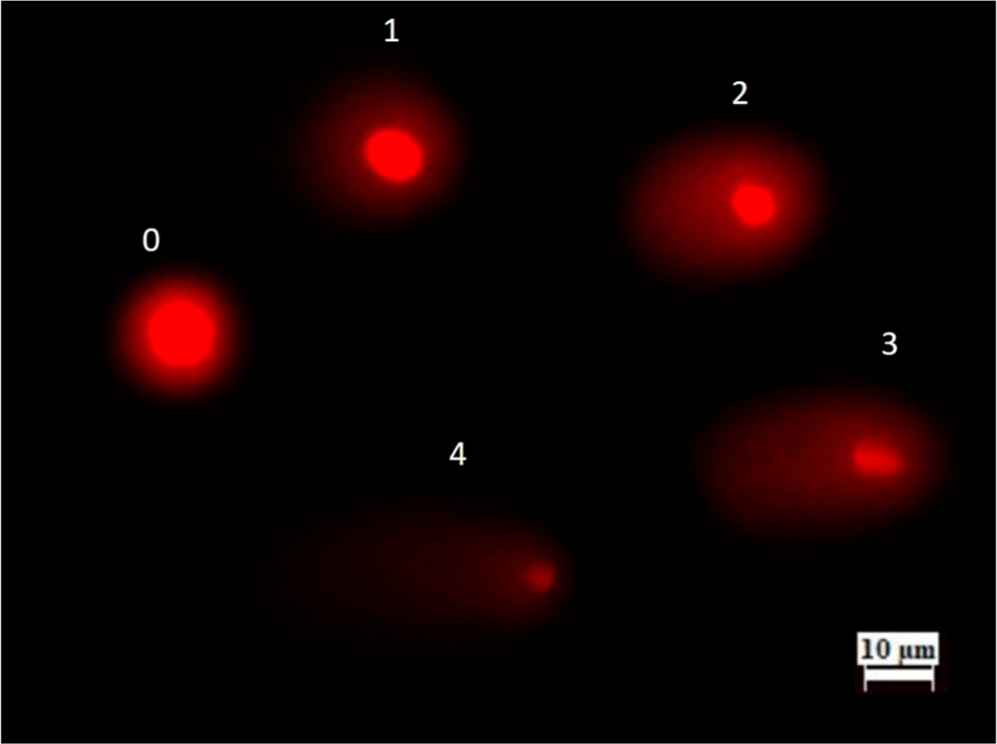
Comet assay scores of DNA damage in *A. cepa* root cells treated with ATO NPs (200×). Scores: 0 = < 5% (none), 1 = 5–20% (slight), 2 = 20–40% (moderate), 3 = 40–85% (severe), 4 = > 85% (extensive).

Ni = Number of cells in *i* degree; *i* = degree of damage (0, 1, 2, 3, 4)

### 2.7 Molecular Docking

#### 2.7.1 Rationale of Receptor Selection

In the experimental part of this study, cyto-genotoxic screening of *A. cepa* root meristematic cells exposed to ATO NPs revealed a steep decrease in the mitotic index (MI), a prominent increase of chromosome laggards, chromosome stickiness, anaphase bridges and polyploid cells, and approximately 27-fold rise of DNA fragmentation in the comet-assay at 100 μg/mL concentration of ATO NPs. These genotoxic effects induced by ATO NPs in *A. cepa* root cells could point to two mechanistic axes: (1) spindle poisoning, (2) direct physicochemical damage to DNA molecules. We therefore selected the tubulin-colchicine complex (hetero 6-mer) (PDB ID: 4O2B, resolution: 2.30 Å) as the primary cytoskeletal target since the microtubule destabilization could provide a molecular-level explanation for the incidence of laggard chromosomes and increased accumulation of mitotic phases in later stages (metaphase, anaphase and telophase) of cell cycle. On the other hand, the synthetic DNA dodecamer d­(CpGpCpGpApApTpTpCpGpCpG)_2_ duplex (PDB ID: 1BNA, resolution: 1.90 Å) was chosen as a target receptor for genomic insult caused by ATO NPs, allowing assessment of DNA binding strength and groove-binding preferences of ATO NPs that might underlie the significant increase of the DNA fragmentation in the comet assay. These two target structures which may reveal the mechanisms of cyto-genotoxic responses detected in vivo, are highly conserved across taxonomic kingdoms of living entities, and constitute high quality 3D structures for modeling of ATO-NPs binding conformations and affinities against these biomacromolecular targets.

#### 2.7.2 Construction of the ATO (Sb-Doped SnO_2_) NP Model in VESTA v3.5.8

The rutile phase SnO_2_ unit cell was downloaded in.cif format from the Materials Project (mp-856) (https://next-gen.materialsproject.org/materials/mp-856) and opened in VESTA program (v3.5.8).^37^ Using Edit → Edit Data→ Unit cell, the crystal symmetry was removed to avoid symmetry-replicated substitutions. A 2 × 2 × 2 supercell was then generated via “Transformation matrix” window with the Rotation matrix *P* set to (2 × 2 × 2); after that the option “Search atoms in the new unit-cell and add them as new sites” was selected so that atomic positions were explicitly created. After wrapping sites into the cell, the resulting supercell contained 16 Sn and 32 O atoms. In the SnO_2_ structure, the pristine unit cell contains 2 Sn atoms and 4 O atoms. Constructing a 2 × 2 × 2 supercell increases the unit-cell volume 8-fold, yielding a supercell with 48 atoms. Then, the supplier’s certificate of analysis (COA) stoichiometry (SnO_2_:Sb_2_O_3_ = 90:10, by weight) result was converted to a molar basis, corresponding to ∼10.3% Sb in the cation fraction. Given the 2 × 2 × 2 lattice (16 cation sites), the closest realizable composition was 2 Sb substitutions (12.5 at %). Thus, the doping concentration in the supercell occurs at 12.5% in all Sn atoms. This corresponds to an Sb doping concentration of approximately 4.17% within a 48 atom supercell configuration. Two Sn sites were therefore replaced by Sb (Structure parameters → select Sn → Symbol→ Sb) using a substitutional Sb­(V) model without introducing oxygen vacancies. To minimize dopant–dopant interactions under periodic boundary conditions, the two substitutions were placed far apart—e.g., near the fractional coordinates (0, 0, 0) and (0.5, 0.5, 0.5)—and the shortest L_Sb_–L_Sb_ distance was verified to be ≥6–8 Å using the Distance tool. The lattice topology and Sb–O octahedral coordination were preserved; no Sb–Sn bonds were defined (metal–oxygen network maintained). The doped supercell was exported as.pdb file, then a short relaxation in Avogadro^38^ (v1.2.0) was performed with the Universal Force Field (UFF) using steepest-descent minimization (500 steps) only to remove close contacts while preserving cell dimensions. The final ATO NP structure was saved in.mol2 format for subsequent docking calculations.

#### 2.7.3 Retrieval of Ligand and Protein Structures and Molecular Docking

In the tubulin docking, colchicine—the cocrystallized reference inhibitor—was obtained by extracting it from its crystal complex. For the nucleic acid docking experiments, MMS, used as a positive control in the experimental section, was retrieved from PubChem (CID: 4156) in.sdf format. Both reference ligands were subjected to the same UFF/steepest-descent minimization protocol in Avogadro^38^ that was used for the ATO NP prior to docking.

In this study, molecular docking experiments were conducted using AutoDock (version 4.2.6)^39^ to explore the molecular interactions of ATO NP against the colchicine binding site of tubulin complex (PDB ID: 4O2B) and the synthetic DNA dodecamer (PDB ID: 1BNA) downloaded from RCSB Protein Data Bank (https://www.rcsb.org/). AutoDock Tools 1.5.7 was employed to merge all nonpolar hydrogen atoms, whereas polar hydrogens on the receptors, ATO NP, MMS, and colchicine were preserved throughout the docking setup. Kollman charges were applied to each receptor, while Gasteiger charges were assigned to the ATO NP, MMS, and colchicine. Docking simulations were performed using a semiflexible protocol in which all ligand torsions were treated as freely rotatable, whereas the receptor structures were maintained rigid. For the tubulin complex, the grid box dimensions were fixed at 60 Å × 60 Å × 60 Å (*x* = 16.22 Å, *y* = 79.39 Å, *z* = 42.58 Å). For the DNA receptor, the grid box was configured as 60 Å × 60 Å × 120 Å (*x* = 14.77 Å, *y* = 20.97 Å, *z* = 8.80 Å). These grid parameters were selected to encompass, respectively, the colchicine-binding pocket of tubulin and the entire accessible molecular surface of the DNA duplex. The tubulin binding site was determined by extracting the coordinates of the cocrystallized inhibitor colchicine.

Binding affinity (docking scores) values (Δ*G* = kcal/mol) obtained for the tubulin–colchicine and DNA–MMS complexes were used as positive control values for relative comparison with the docking results of ATO NP against the same two targets. Since the default atom-type library of AutoDock 4.2.6 does not include antimony (Sb) and tin (Sn), we used a community-extended *AD4_parameters.dat* obtained from the AutoDock Web site^40–43^ to add these atom types. The modified parameter file was then explicitly referenced by the grid (.gpf) and docking (.dpf) input files. Before docking, the ligands (ATO NP, MMS, and colchicine) and the receptors (tubulin heterodimer and DNA duplex) structures were converted to.PDBQT format using AutoDock Tools 1.5.7.^44^ Full atomic parameters of.gpf and.dpf files are given in Supporting Information.

Each molecular docking simulation was executed with 20 independent genetic algorithm (GA) runs, a maximum of 5.000.000 energy evaluations, and 27.000 maximum number of generations. Mutation and crossover rates were assigned as default values of 0.02 and 0.8, respectively. Upon completion of the 20 GA runs, each binding mode predicted for ATO NP, MMS, and colchicine against each receptor (tubulin heterodimer and DNA) was clustered by root-mean-square deviation (RMSD) and ranked according to the most favorable (most negative) docking score (Δ*G* = kcal/mol). The top-ranked receptor–ligand conformations were then rendered and qualitatively analyzed in BIOVIA Discovery Studio Visualizer v16.^45^

### 2.8 Statistical Analysis

Data were expressed as mean ± standard deviation and analyzed using one-way analysis of variance (ANOVA), followed by Duncan’s multiple range test for posthoc comparisons at a significance level of *P* < 0.05. Dose-dependent effects were assessed using Pearson correlation analysis (*P* < 0.01), and all statistical evaluations were performed using SPSS software version 23.0.

## 3 Results and Discussion

### 3.1 *A. cepa* Cyto-Genotoxicity Assay

#### 3.1.1 *A. cepa* Ana-Telophase Test

Four concentrations of the ATO (12.5, 25, 50, or 100 μg/mL) were applied to Allium root tips for 4 h in order to examine the effects on MI and CAs. MI was shown to be considerably reduced in the ATO NPs-treated groups (15.15 ± 0.71 to 13.99 ± 0.58; Table 1; F_5, 24_ = 83.189, *p* < 0.001) in a dose-dependent manner as compared to the control (16.06 ± 0.42). There was a strong negative correlation between group and MI (r (18) = −0.654, *p* = 0.002). A marked decrease in prophase frequency and a corresponding increase in other mitotic phases suggest that ATO NPs exert strong antiproliferative effects. This inhibition may result from multiple cytotoxic mechanisms. ATO NPs are known to induce reactive oxygen species (ROS), causing DNA damage and activating checkpoint pathways that arrest the cell cycle at the G2/M phase.^23,46^ They may also disrupt spindle microtubule organization, leading to chromosomal missegregation or metaphase arrest.^47,48^ Moreover, interference with key cell cycle regulatory proteins and inhibition of DNA polymerase and other essential enzymes could contribute to the observed antimitotic outcomes.^49,50^ However, the toxicity of ATO and related NPs appears to be highly dependent on concentration, exposure duration, and biological model. For instance, Park et al.^16^ found that ATO NPs up to 1000 μg/mL had no adverse effects on *Brassica campestris* seed germination, plant growth, or *Caenorhabditis elegans* survival. Similarly, *Macrotyloma uniflorum* seeds treated with SnO_2_ NPs (100 μg/mL) exhibited increased root and shoot lengths compared to controls.^51^ In mammalian systems, ATO NPs (≤100 μg/mL) did not significantly affect J774A1 macrophage viability after 24 h^14^ and only minor cytotoxicity was observed in 3D human bronchial epithelial cultures exposed to aerosolized ATO NPs.^52^ Bregoli et al.^53^ reported that Sb_2_O_3_ NPs inhibited the proliferation of human erythroid progenitor cells through membrane–level interactions without cellular accumulation. Similarly, Titma et al.^54^ found no acute toxicity in A549 cells exposed to Sb_2_O_3_ NPs (3–100 μg/mL) for 24 h, although prolonged exposure for up to 9 days significantly increased toxicity, with an EC_50_ value of 22 μg/mL.

**Table 1:** Mitotic Index and Phase Distribution in *A. cepa* Root Tips After 4 h Exposure to ATO NPs^a^

concentration (μg/mL)	CCN	MI ± SD	mitotic phases (%) ± standard deviation (SD)^a^				
				prophase	metaphase	anaphase	telophase
control	-	5264	16.06 ± 0.42a	56.18 ± 1.48a	8.89 ± 1a	8.15 ± 0.83a	26.78 ± 1.64a
MMS	10	6117	9.73 ± 0.47b	44.82 ± 2.72b	9.54 ± 1.91a	12.77 ± 1.68b	32.87 ± 1.39b
ATO NPs	12.5	5980	15.15 ± 0.71c	38.27 ± 2.23c	13.89 ± 2.17b	11.43 ± 1.85b	36.42 ± 0.58 cd
	25	5712	14.97 ± 0.35 cd	37.39 ± 1.13c	12.52 ± 1.61b	12.22 ± 2.63b	37.87 ± 2.62d
	50	5768	14.29 ± 0.66de	41.36 ± 1.96de	13.08 ± 0.87b	10.55 ± 1.27b	35.01 ± 2.63bc
	100	5966	13.99 ± 0.58e	35.03 ± 1.14e	14.42 ± 1.12b	12.48 ± 1.33b	38.07 ± 1.50d

a Means with the same letter for each column do not differ statistically at the level of 0.05. CCN: Counting cell number.

Figures 2 and 3 present the CAs observed in *A. cepa* ana-telophase cells following treatment with ATO NPs. A significant concentration-dependent increase in total CAs (r (18) = 0.967 *p* < 0.001) was noted alongside a concomitant decrease in the MI. The percentage of CAs recorded were 4.2 ± 0.84% (negative control), 19.6 ± 1.14% (positive control, MMS), and 9.2 ± 1.1%, 13 ± 0.71%, 16.2 ± 0.84%, and 18.6 ± 1.14% at 12.5, 25, 50, and 100 μg/mL of ATO NPs, respectively. Chromosome laggards and stickiness were the most frequent types of aberration, especially at 50 and 100 μg/mL, indicating that ATO NPs may interfere with chromatin condensation and spindle organization. The presence of chromosome stickiness, polyploidy, and disturbed ana-telophase figures at higher concentrations further suggests that ATO NPs disrupt normal chromosomal segregation, potentially through alterations in microtubule stability or kinetochore function. Disruption of microtubule dynamics, deformation of the mitotic spindle apparatus, or the inability of chromosomes to migrate properly toward opposite poles during anaphase could be the cause of chromosomal laggards (Figure 3e).^22,23,46^ Stickiness (Figure 3f) may arise from abnormal chromatin condensation, extrachromosomal entanglement of chromatin fibers, inappropriate protein–protein interactions, partial degradation of nucleoproteins, or DNA depolymerization—all of which may result in mitotic arrest and cell death.^26,55,56^ Anaphase bridges (Figure 3g) may arise from structural anomalies such as the fusion of chromatids or chromosomes, the presence of dicentric chromosomes, malfunctioning replication processes, or sustained chromosomal adhesion—each pointing to a clastogenic response.^57,58^ Polyploidy (Figure 3h) induction is thought to be caused by inhibiting cytokinesis or interfering with mitotic spindle components during chromosomal segregation.^59,60^ Overall, the increasing frequency of CAs and the reduction in MI provide strong evidence that ATO NPs exert both clastogenic and cytotoxic effects on *A. cepa* root meristem cells. These effects likely arise from direct interactions of NPs with DNA or indirect oxidative stress–mediated damage leading to chromosomal instability. CAs such as chromosome stickiness and anaphase bridges observed in *A. cepa* cells after ATO NPs exposure are consistent with earlier findings on other metal oxide nanoparticles. SnO_2_ NPs have similarly been reported to induce mitotic CAs, including sticky chromosomes and anaphase bridges.^51^ In contrast, in vivo studies indicate that antimony trioxide exhibits minimal genotoxic potential in mammalian systems. Oral administration of Sb_2_O_3_ to rodents, both acutely and repeatedly at doses near the maximum tolerated level, did not produce significant chromosomal alterations in bone marrow cells.^61^ This discrepancy between plant-based assays and animal studies underscores the importance of biological context: factors such as exposure route, nanoparticle bioavailability, and metabolic processing can markedly influence genotoxic outcomes. Thus, while ATO NPs can disrupt mitosis and induce chromosomal instability in rapidly dividing plant cells, their genotoxic risk under physiological mammalian exposure conditions appears comparatively low.

**Figure 2:**
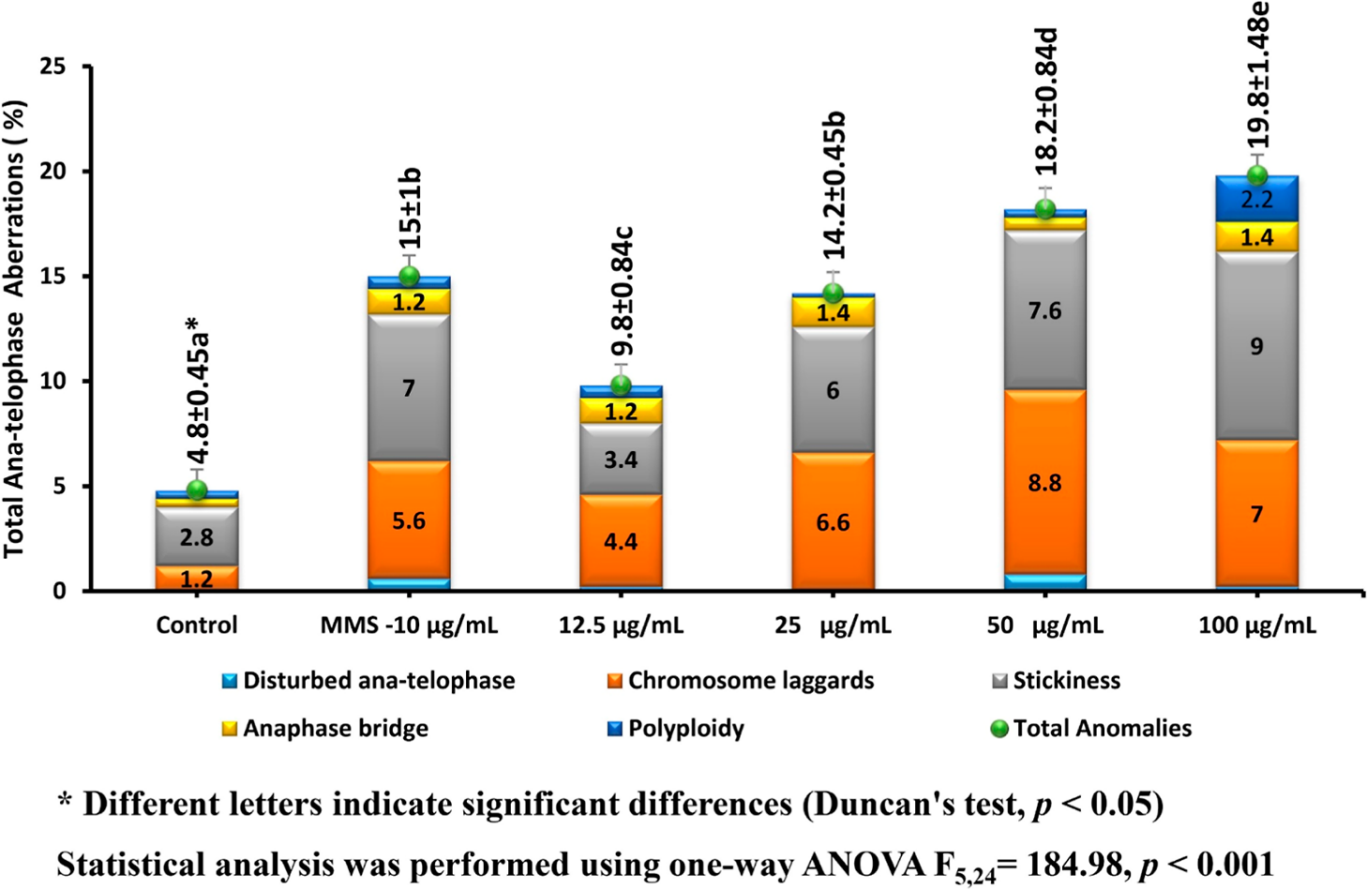
Effect of ATO NPs on CAs in *A. cepa* root tips. The results for each column represent the meaning of five replicates, along with the standard deviations (±) for total aberrations.

**Figure 3:**
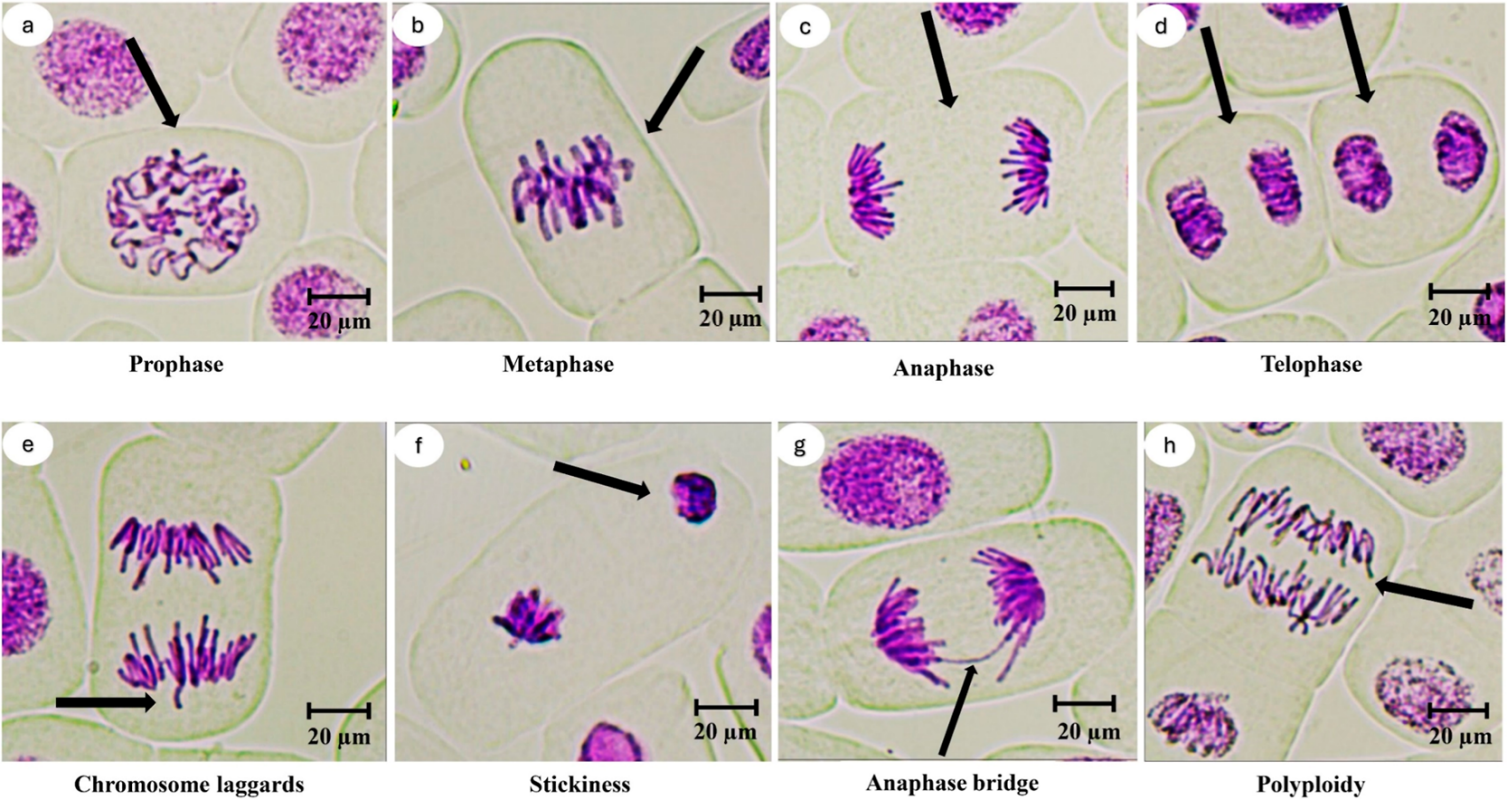
Representative microphotographs showing normal mitotic phases and CAs in *A. cepa* root tip cells exposed to ATO nanoparticles. (a) Prophase, (b) Metaphase, (c) Anaphase, and (d) Telophase represent normal mitotic phases. (e–h) illustrate common CAs observed after ATO NPs exposure: (e) chromosome laggards, (f) stickiness, (g) anaphase bridge, and (h) polyploidy. Arrows indicate the specific mitotic stages and CAs within the meristematic cells (400×, scale bar: 20 μm).

#### 3.1.2 SEM and TEM Analysis on Allium Roots

In figure SEM can be seen that, compared to untreated control group (Figure 4A–C), ATO NPs-treated onion roots presented thinner roots with undefined areas of growth and absorption (Figure 4D,E and inset). Cells of the root tips were smaller and suffered more from dehydration (Figure 4F). The EDX analyses (Figure 4G,H) did not evidentiate the presence of Sb in the root’s samples, but that may be due to the low concentration of the NPs on the surface of the roots tissue, which probably were below 0.1 wt %, which is the limit of detection of this analysis.

**Figure 4:**
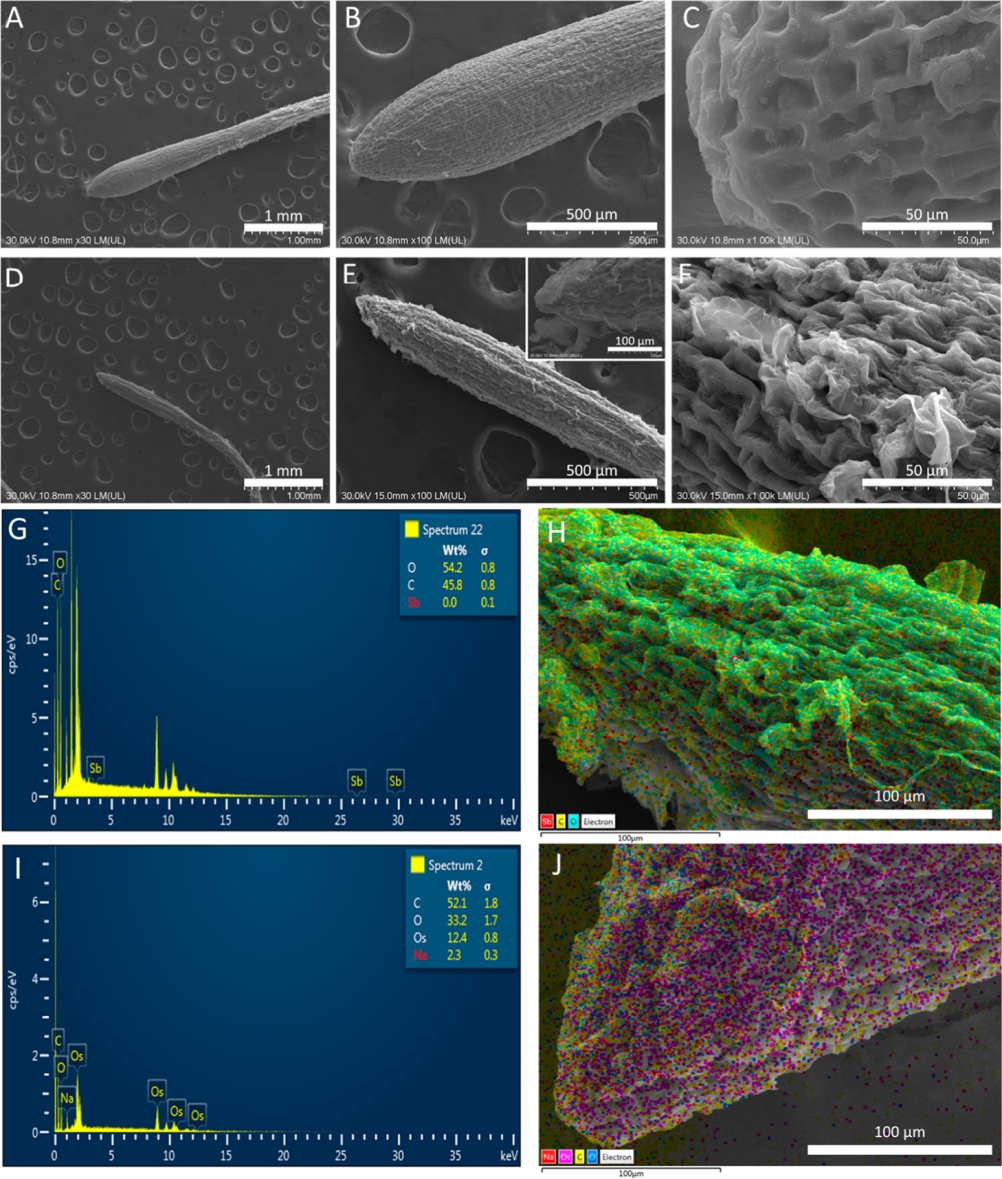
SEM images of untreated (A–C) and ATO treated (D–F) *A. cepa* roots. EDX analysis and mapping (G&H) of ATO-treated roots.

In Figure 5D–F, the presence of the NPs can be observed. The ATO NPs were absorbed by the root cells and were distributed throughout the cell, mostly accumulating in the vacuoles, as indicated by the blue arrows. NPs can also be seen freely in the cytoplasm (Figure 5F) or attached to the mitochondrial membrane (Figure 5E). Their presence is possible due to the electrondense nature of their composition, and they seem to form structures or aggregates of various dimensions (50–300 nm) and may be in association with other molecular species from the cell (proteins or lipids) that are less electrondense. EDX point analysis (Figure 5G) confirmed the ATO origin of the electrondense material through the presence of Sn (1.2 ± 0.1 wt %), although the original shape of the NPs was not retained any longer. Sb was not detected in any of the analyzed root sections. Compared to the untreated control (Figure 5A–C) where cells presented large vacuoles and thick cell walls, the ATO treated roots presented many small vacuoles (Figure 5A), thin cell walls (Figure 5C) and mitochondria were affected. In Figure 5B, it can be observed that the vacuole formed an accumulation of membrane folds inward, which is a sign of dehydration.

**Figure 5:**
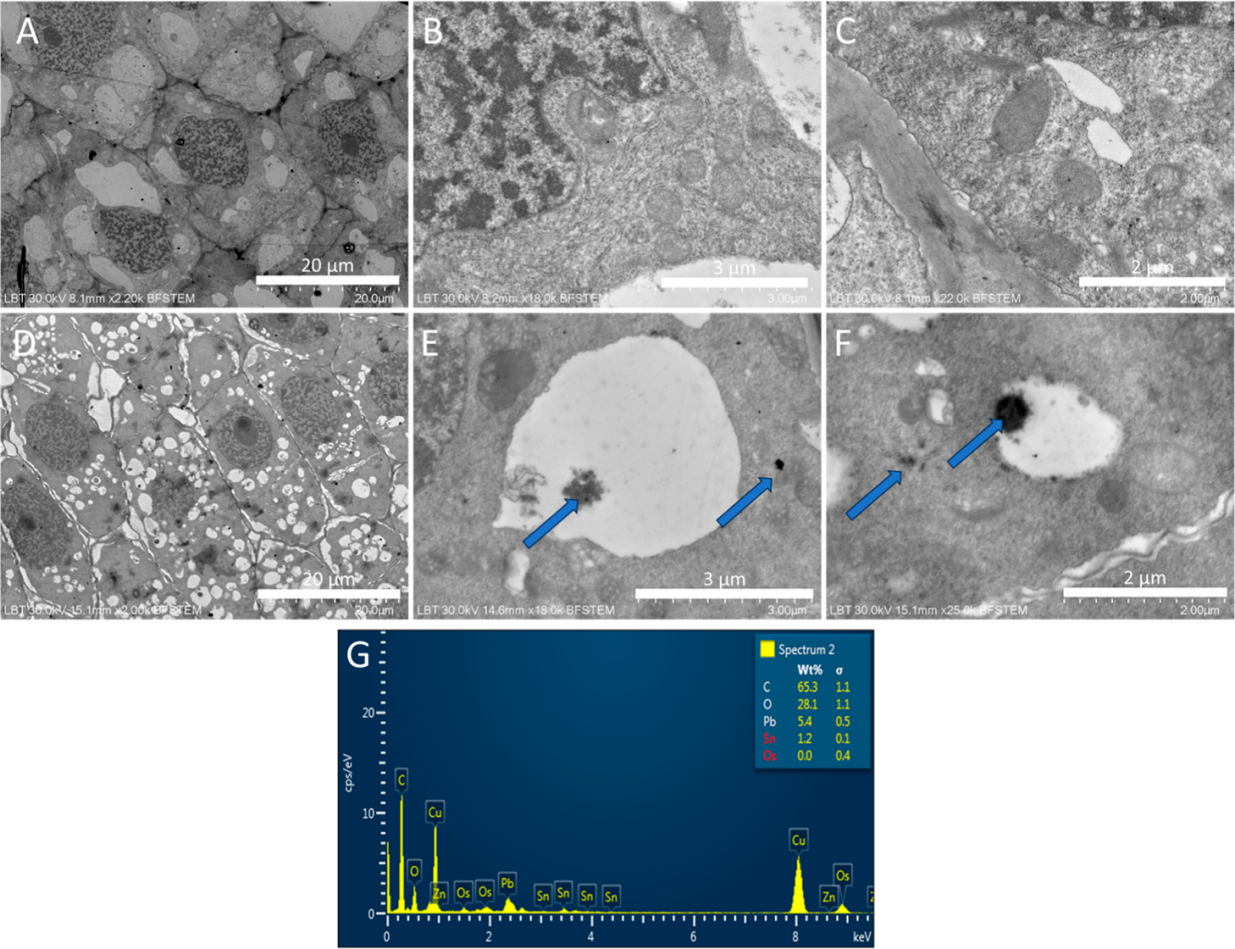
TEM micrographs of control onion roots (A–C) and ATO-treated onion roots (D–F). EDX analysis of an electrondense inclusion shows Sn, indicating ATO origin and therefore ATO uptake (G). Arrows indicate ATO inclusions in the root cells, bars represent 20, 3, and 2 μm.

#### 3.1.3 Comet Assay

The comet assay results (Figure 6) illustrate the genotoxic effects of ATO NPs on *A. cepa* root meristem cells, quantified using a five-point visual scoring scale (0–4) corresponding to the extent of DNA migration. As shown in Figure 6, the control group exhibited primarily Class 0 nuclei, confirming genomic integrity in unexposed cells. In contrast, cells treated with the positive control, methylmethanesulfonate (MMS, 10 μg/mL), displayed a significant increase in Classes 3 and 4, yielding the highest total DNA damage (157.33 ± 5.86 AU). A similar but concentration-dependent pattern was observed in ATO NP–treated groups (r(10) = 0.953 *p* < 0.001). The total DNA damage increased markedly from 5.67 ± 1.53 AU at 12.5 μg/mL to 74.67 ± 4.93 AU, 120.67 ± 2.52 AU, 133.33 ± 2.08 AU, and 154.33 ± 3.51 AU at 25, 50, and 100 μg/mL, respectively. At lower concentrations (12.5 μg/mL), most nuclei remained within Classes 0–2, suggesting limited DNA fragmentation and partial repair capacity. However, at ≥ 25 μg/mL, the proportion of Classes 3 and 4 comets increased substantially, indicating extensive single- and double-strand breaks. The strong, concentration-dependent rise in total DNA damage reflects a direct correlation between ATO NP exposure and genotoxic potential. Statistical analysis confirmed that the differences among the treatments were significant (*p* < 0.05), except between 100 μg/mL ATO NPs and the positive control, which did not differ statistically—implying comparable DNA-damaging capacity. Similar increases in DNA damage have been reported with other types of antimony and/or tin-based NPs. The concentrations of the SnO_2_ NPs were shown to correlate with the higher amounts of malondialdehyde reported in *A. cepa* roots. The cytotoxicity demonstrated by the concentration of 100 μg/mL was relatively lower than that obtained at higher doses, according to the cell death experiment.^51^ A significant induction in DNA damage was observed by the Comet assay in SKOV3 cells exposed to SnO_2_ NPs and folic acid (FA)-decorated tin oxide NPs (FA-SnO_2_ NPs) and on pulmonary A549 cells exposed to indium-doped SnO_2_ (ITO) due to over production of ROS, respectively.^62,63^ The production of ROS and malondialdehyde was significantly (*P* < 0.05) elevated by two typical Sb­(III) substances, antimony trioxide NPs and its soluble Sb­(III) counterpart antimony potassium tartrate, in comparison to the control group on *Daphnia magna* indicating that both Sb­(III) compounds induced membrane damage and oxidative stress.^64^ Unlike our study, there are also studies that give negative or insignificant results. According to El Shanawany et al.,^65^ oxidative stress was not the cause of the genotoxic effects of Sb_2_O_3_ on occupationally exposed workers. After being exposed to the ATO NPs on MucilAir cultures, there were no discernible changes in the levels of primary (strand breaks) and oxidative (FPG-sensitive sites) DNA damage as compared to the controls.^52^

**Figure 6:**
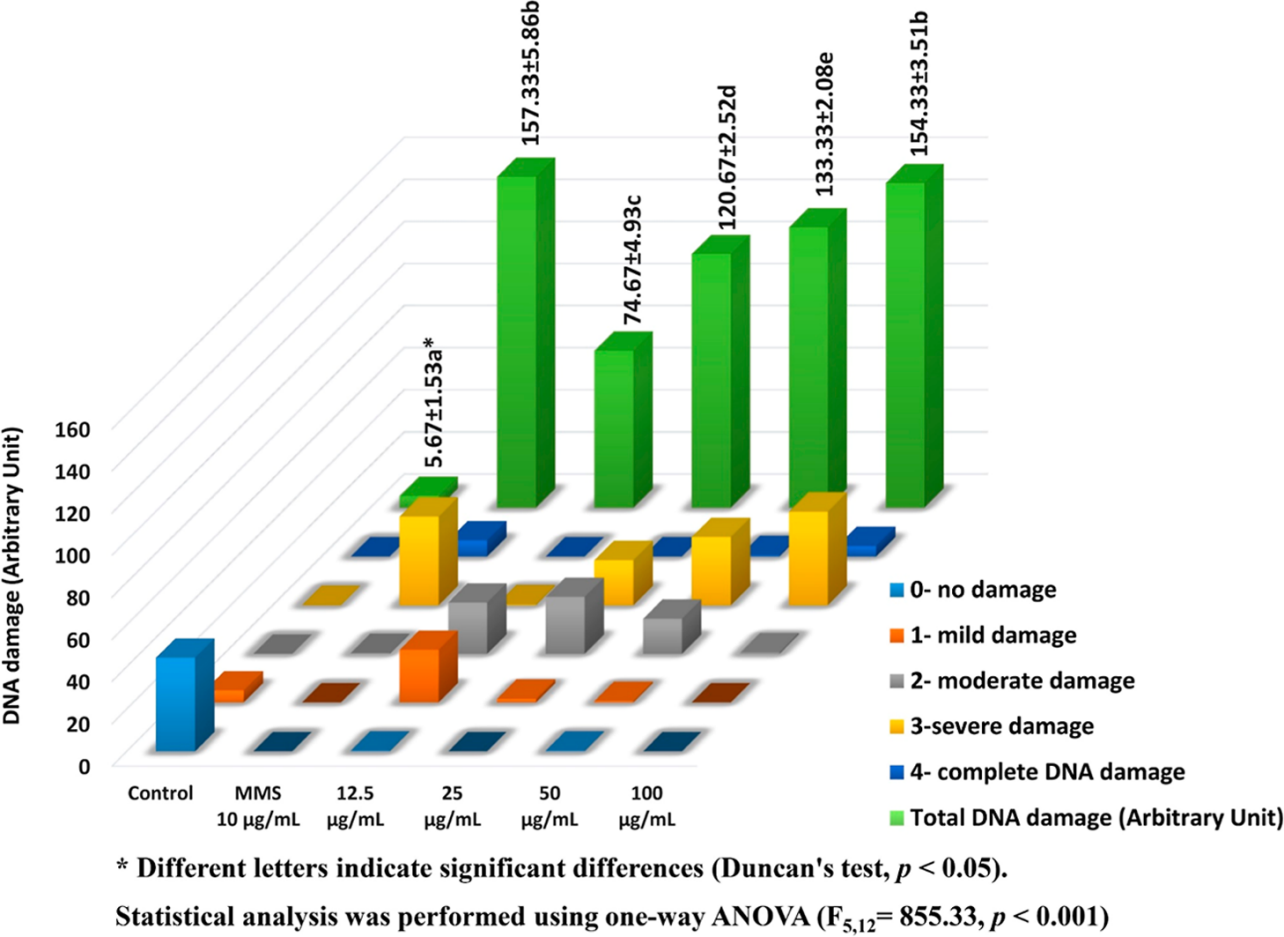
Assessment of DNA damage in *A. cepa* root tip cells following ATO NPs exposure after 4 h. The results for each column represent the meaning of three replicates, along with the standard deviations (±) for total DNA damage.

### 3.2 Molecular Docking

Colchicine—the cocrystallized reference inhibitor—exhibited an energetically favorable binding within the colchicine-binding pocket of the tubulin heterodimer (Δ*G* = −9.43 kcal/mol). The intermolecular interaction network comprised two C–H bonds with Val746 and Asp868; extensive hydrophobic contacts with Lys691, Leu679, Leu685, Ala687, Leu692, Ile749, Ala747, and Ala785; and a π–sulfur interaction with Met696 (Table 2). The ATO nanoparticle (NP) exhibited an energetically highly favorable binding affinity within the colchicine-binding pocket of the tubulin heterodimer (Δ*G* = −11.06 kcal/mol). The intermolecular interaction network comprises four hydrogen (H) bonds with residues Gln15, Tyr224, and Thr225; four C–H bonds with Thr223 and Thr225; and one π–sulfur interaction with Tyr224 (Table 2 and Figure 7).

**Table 2:** Predicted Docking Score (Δ*G*: Kcal/mol) and Interaction Profiles of Colchicine and ATO NP within the Colchicine-Binding Pocket at the Interface of the α/β-Tubulin Heterodimer^a^ ^,^ ^b^

compound	molecular weight (g/mol)	receptor^a^ (4O2B)	Δ*G*_best_ (kcal/mol)^b^	classical H-bond	nonclassical H-bond (carbon–hydrogen)	hydrophobic alkyl/π-alkyl interaction	halogen (pi-sulfur)
colchicine (cocrystallized inhibitor)	399.44	α/β-tubulin	—9.43	—	Val746 (3.38 Å), Asp868 (3.41 Å)	Lys691 (5.43 Å), Leu679 (4.37 Å), Leu685 (3.89 Å, 5.16 Å), Ala687 (4.41 Å, 4.60 Å), Leu692 (3.91 Å, 4.61 Å, 4.85 Å, 5.49 Å), Ile749 (4.59 Å), Ala747 (4.24 Å), Ala785 (4.13 Å)	Met696 (5.13 Å)
ATO NP	1118	α/β-tubulin	—11.06	Gln15 (3.37 Å), Tyr224 (2.07 Å, 2.89 Å), Thr225 (1.76 Å)	Thr223 (1.95 Å, 2.06 Å, 2.81 Å), Thr225 (2.72 Å)	—	Tyr224 (5.26 Å)

a PDB ID: 4O2B = Structure of the tubulin-colchicine complex; https://www.rcsb.org/structure/4O2B.

b Δ*G*_best_: Docking score (kcal/mol) of the most favorable ligand pose.

**Figure 7:**
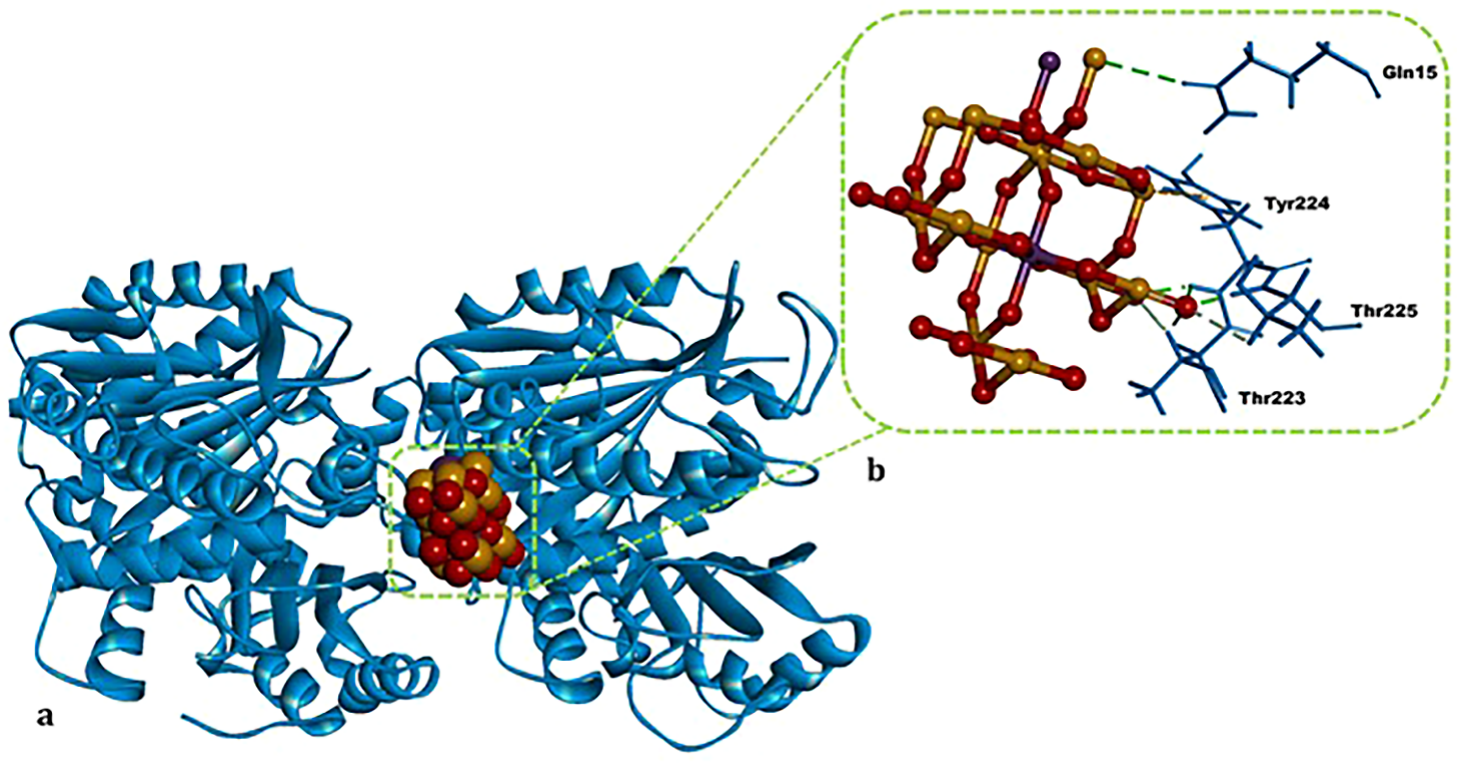
Top-ranked postdocking conformation of ATO NPs bound at the colchicine-binding site of the human α/β-tubulin heterodimer (PDB ID: 4O2B). (a). Overall three-dimensional representation of the α/β-tubulin–ATO NPs complex. (b). Enlarged view of the ATO NPs within the colchicine-binding pocket. Green dashed lines denote hydrogen (H) bonds, while bold black labels indicate residue labels and nonbonded interaction distances (Å). The figure was rendered with BIOVIA Discovery Studio Visualizer v16.

Within the tubulin heterodimer’s colchicine-binding pocket, the ATO NP yielded a more favorable docking score (Δ*G* = −11.06 kcal/mol) than colchicine (Δ*G* = −9.43 kcal/mol)—a preliminary finding that could be consistent with the observed decrease in mitotic MI (Table 1).

MMS—the experimental DNA mutagen—exhibited a modestly favorable docking score within the minor groove of B-DNA (Δ*G* = −3.80 kcal/mol). The intermolecular interaction network of MMS comprised conventional H-bonds with Gua10 and Gua16; a C–H bond with Gua16; and a π–sulfur interaction with Gua16 (Table 3). The ATO NP, in its energetically most favorable pose, engaged the major groove of dsDNA, forming conventional H-bonds with Ade5, Ade6, Gua16, Ade17 and Ade18; additional C–H bonds with Ade5 and Gua16; and a π-donor H-bond with Gua16 (Table 3 and Figure 8). The predicted docking score for ATO NP binding to dsDNA is highly favorable (Δ*G* = −12.07 kcal/mol; Table 3). Against B-DNA, the ATO NP likewise showed a more favorable docking score (Δ*G* = −12.07 kcal/mol) than MMS (Δ*G* = −3.80 kcal/mol), an exploratory result that may be qualitatively consistent with the increased CAs frequency observed in *A. cepa* root tip cells (Figure 2); stable ATO NP–DNA contacts could stall the replication machinery during S phase and thereby contribute to DNA damage.

**Table 3:** Predicted Docking Score (Δ*G*: Kcal/mol), Preferred Binding Mode, and Nucleotide Interaction Details with Corresponding Non-Bonded Distances (Å) for Methylmethanesulfonate (MMS) and ATO NP Against Double-Stranded (ds) DNA^a^

compound	molecular weight (g/mol)	receptor^a^ (1BNA)	Δ*G* best (kcal/mol)[Table-fn t3fn3]	binding mode	classical H-bond	Nonclassical H-bond (carbon–hydrogen, pi-donor)	miscellaneous (pi-sulfur)
MMS (positive control)	110.13	B-DNA	—3.80	Minor groove	Gua10 (1.86 Å), Gua16 (1.72 Å, 1.97 Å)	Gua16 (3.64 Å)	Gua16 (5.08 Å)
ATO NP	1118	B-DNA	—12.07	Major groove	Ade5 (2.47 Å, 2.79 Å, 2.87 Å, 3.16 Å), Ade6 (1.96 Å, 2.24 Å, 3.08 Å, 3.30 Å, Gua16 (2.68 Å), Ade17 (1.74 Å), Ade18 (1.84 Å)	Ade5 (2.62 Å), Gua16 (3.51, 4.01)	—

a PDB ID: 1BNA = Structure of a B-DNA dodecamer; https://www.rcsb.org/structure/1BNA.

MMS: methylmethanesulfonate – experimental positive control.

b Δ*G*_best_: Docking score (kcal/mol) of the most favorable ligand pose.

**Figure 8:**
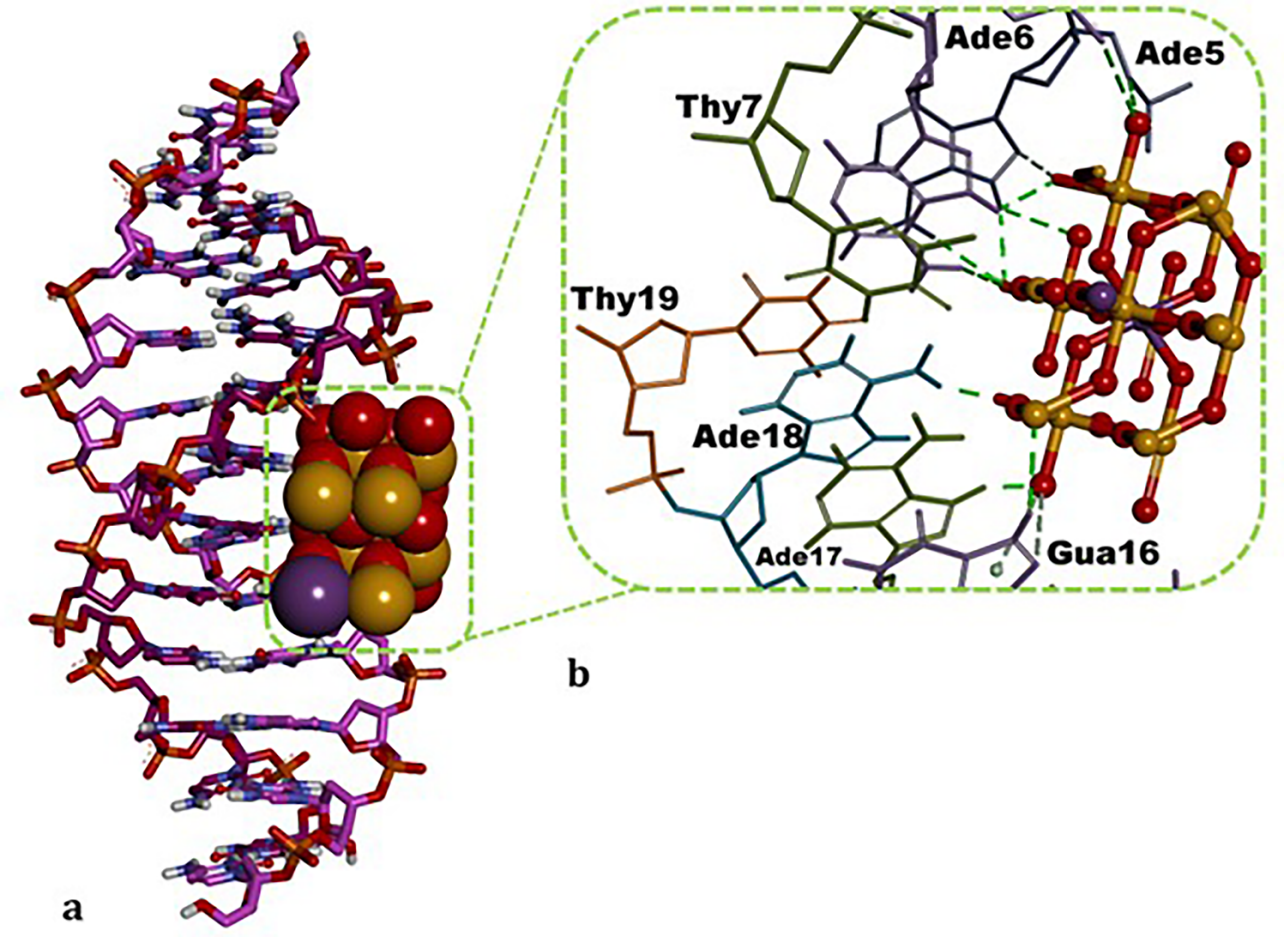
Binding mode of ATO NP within the B-DNA dodecamer duplex (PDB ID: 1BNA). (a). Three-dimensional representation of the DNA—ATO NP complex, showing the DNA receptor in stick mode and ATO NP in Corey–Pauling–Koltun (CPK) style. Note that the ATO NP snugly occupy the major groove. (b). Enlarged interaction diagram highlighting contacts between ATO NP and individual DNA nucleotides. Green dashed lines denote conventional hydrogen (H) bonds, and light-green dashed lines denote C–H and π-donor H-bonds; nucleotide identifiers are labeled in black. The figure was generated with BIOVIA Discovery Studio Visualizer v16.

ATO NPs adopted the energetically most favorable pose in the minor groove of dsDNA, where they were stabilized by five H-bonds formed with Ade6, Thy7 and Thy19 (Figure 8). The nucleotides interacted by ATO NPs within the double-stranded (ds) synthetic B-DNA dodecamer—together with their binding mode and affinity—are summarized in Table 3. Notably, the predicted docking score of ATO NPs against dsDNA (Δ*G* = —5.63 kcal/mol) is markedly more favorable than that of the experimental DNA-alkylating agent methylmethanesulfonate (MMS) (Δ*G* = —3.80 kcal/mol) (Table 3), suggesting that ATO NPs may induce DNA damage by forming stable DNA–ligand complexes during the DNA replication phase.

In computational molecular docking, we obtained initial, hypothesis-generating indications of how ATO NP may interact with tubulin and DNA, providing a preliminary context for the experimentally observed mitotic perturbation and DNA damage. Docking predicted energetically highly favorable interactions with both the tubulin heterodimer (Table 2 and Figure 7) and the major groove of a synthetic DNA fragment (Table 3 and Figure 8). Although the genotoxic mechanisms of ATO NPs remain insufficiently characterized, tin oxide (SnO_2_) nanoparticles—the principal constituent of ATO—have been reported to induce particle size–and dose–dependent cytotoxicity in HCT116 and A549 cancer cells via reactive oxygen species–mediated oxidative stress.^66^ While those literature data are consistent with an indirect, ROS-mediated pathway, our docking study suggests that ATO NPs may also interact with tubulin—a central regulator of mitosis—and with DNA. This dual, energetically favorable affinity of ATO NP against these biomacromolecules points to a potentially multimodal genotoxicity (involving mitotic perturbation and a possible DNA interaction) and warrants further molecular investigation. However, the docking results for the ATO NP reported in this study indicate only candidate interaction poses with two biomolecules. Because NPs are materials with extended surfaces and collective physicochemical properties rather than small molecules, the predictive docking results presented here should not be interpreted as quantitative measures of binding affinity; rather, they generate testable hypotheses for follow-up studies.

## 4 Conclusions

The high structural homogeneity and crystallinity of ATO NPs were confirmed through HRTEM, EDX, and XRD characterization. This study demonstrates that ATO NPs induce significant cyto-genotoxic effects in *A. cepa* root meristematic cells, evidenced by a concentration-dependent decrease in mitotic activity, elevated CAs frequencies, and increased DNA damage. The cytological and genotoxic end points observed were further supported by SEM analysis, which revealed structural alterations at the cellular level. Complementary in silico docking provided preliminary data that ATO NPs may favorably interact against both the colchicine-binding site of tubulin and DNA. These initial observations are consistent with a possible dual genotoxic mode involving microtubule perturbation and potential DNA interaction, warranting further investigation such as tubulin polymerization kinetics and DNA-binding assays. Although Allium and comet assays revealed genotoxic effects, the absence of uptake data, ionic controls, and dispersion analysis limits causal interpretation. The 4 h exposure was chosen based on standard *A. cepa* protocols to detect early cytogenetic responses. While suitable for preliminary screening, extended exposures (e.g., 24–72 h) would help determine effect persistence and improve environmental relevance. Overall, the integration of experimental and computational approaches underscores the potential risk associated with ATO NPs exposure and highlights the necessity for further toxicological evaluations in more complex biological systems.

## Supplementary Material



## Notes

Characterization data of ATO NPs; XRD analysis, HRTEM and EDX results, zeta potential, particle size distribution, and dispersion stability measurements. Detailed molecular docking parameters; AutoDock atomic parameters, grid and docking parameter files (.gpf and.dpf), and extended docking setup information for tubulin and DNA targets (PDF)
