# Supplementary material for: Cyto-Genotoxic Impacts of Antimony Tin Oxide (ATO) Nanoparticles on Allium cepa Root Meristem Cells: An Integrative Experimental and in Silico Approach
Source: ACS Omega. 2026 Jan 23;11(5):7532–44. doi: 10.1021/acsomega.5c08687 (PMC12902866; doi:10.1021/acsomega.5c08687)
Supplement: Supplementary file 1 [file ao5c08687_si_001.pdf]

Cyto-genotoxic Impacts of Antimony Tin Oxide (ATO) Nanoparticles on *Allium cepa* Root Meristem Cells: An Integrative Experimental and *In Silico* Approach

Recep Liman[1*], Erman Salih Istifli[2], Yaser Acikbas[3], Yudum Yeltekin Uğur[1], Maria Suciu[4,5], Lucian Barbu-Tudoran[4,5], İbrahim Hakkı Ciğerci[6]

[1] Molecular Biology and Genetics Department, Faculty of Engineering and Natural Sciences, Uşak University, 64300, Uşak, Turkiye

[2] Biology Department, Faculty of Science and Literature, Cukurova University, 01330, Adana, Turkiye

[3] Electrical and Electronic Engineering Department, Faculty of Engineering and Natural Sciences, Uşak University, Usak 64300, Türkiye

[4] National Institute for Research and Development of Isotopic and Molecular Technologies (INCDTIM), 400293 Cluj-Napoca, Romania

[5] Electron Microscopy Center "C. Craciun", Faculty of Biology and Geology, Babes-Bolyai University, 400006 Cluj-Napoca, Romania

[6] Molecular Biology and Genetics Department, Faculty of Science and Literatures, Afyon Kocatepe University, 03200, Afyon, Turkiye

***Recep Liman**
https://orcid.org/0000-0002-7944-4952
Uşak University
Faculty of Engineering and Natural Sciences
Molecular Biology and Genetics Department
1 Eylül Campus
64300-UŞAK
TÜRKİYE
E-mails: rliman@hotmail.com; recep.liman@usak.edu.tr
Tel.: +90 5556214251
Fax: +90 276 221 21 35

Characterization of ATO NPs

X-ray diffraction (XRD) is one of the most important techniques used to characterize important parameters, such as the crystallographic structures of materials, the distances between planes, and the arrangement of lattice atoms. As shown in Fig. S1, the XRD spectrum confirms the presence of SnO_2 in ATO NPs. The XRD peaks obtained were assigned to JCPDS card number 01-072-1147 (SnO_2). Fig. S1 shows the broad peaks that were observed at 2θ values of 26.57°, 33.87°, 37.77°, 51.8°, 54.34°, 57.93°, 61.99°, 65.36°, 71.61°, and 78.71°. These peaks can be ascribed to the tetragonal rutile structure of the SnO_2 crystal, which exhibits a close match with the (1 1 0), (1 0 1), (2 0 0), (2 1 1), (2 2 0), (0 0 2), (3 1 0), (3 0 1), (2 0 2), and (3 2 1) planes of SnO_2, respectively.[1-4] These broad diffraction peaks indicate that the SnO_2 has nanocrystalline particles.

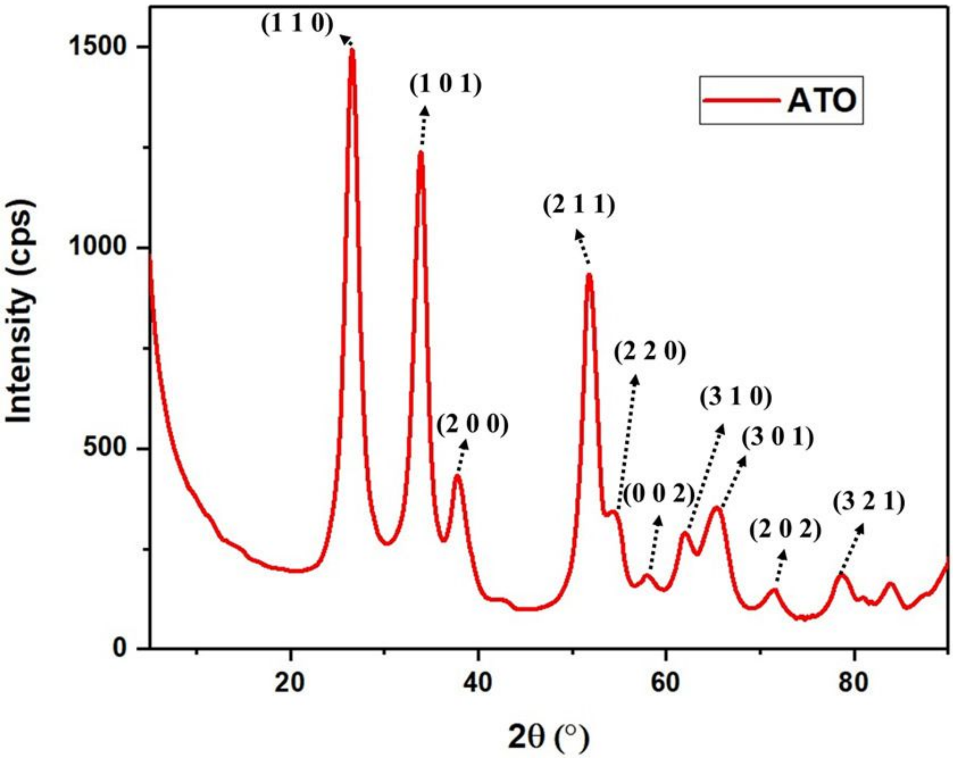

Fig. S1. XRD patterns of ATO NPs. The distinct diffraction peaks indexed to the (110), (101), (200), (211), (220), (002), (310), (301), (202), and (321) planes correspond to the tetragonal rutile structure of SnO_2.

Fig. S2 shows the EDX results, which provide spectral data information for the ATO NPs sample. The inset figures in Fig. S2 show the HRTEM results at 50 and 100 nm scales. Upon examining the HRTEM results, dark-colored rings were observed around the formations in the matrix. The Selected Area Diffraction Pattern technique was used to determine the

structures of these formations. In this context, mixed-type diffraction patterns (dotted ring) and mixed-type diffraction patterns (amorphous + dotted ring) were obtained from a single area. Based on these results, it was determined that the formations have an amorphous and crystalline structure. Furthermore, the EDX results indicate that Sb and Sn exhibit quantitative values in formations with both amorphous and crystalline structures. Additionally, zeta potential, an important parameter for suspension stability, was measured for the ATO NPs sample in this study. Thus, the repulsive or attractive force between the particles in this sample was evaluated. In this context, a greater absolute value of the zeta potential indicates a better dispersion ability. The zeta potential of the ATO NPs sample was measured at -44 mV in distilled water with standard deviation of 5.49 mV and conductivity of ATO NPs was measured as 0.023 mS/cm. Compared to previous studies, this large Zeta potential indicates a strong repulsive force between the electric double layers on the particle surface, suggesting that the ATO NPs are highly dispersible.[5, 6] In addition to zeta potential analysis, the average particle size and polydispersity index (PDI) of the ATO NP dispersion were measured as 142 nm and 0.194, respectively. This indicates a relatively narrow and uniform size distribution and confirms good stability. The slightly higher particle size compared to the expected range may be due to the ATO NPs forming aggregates in the water suspension during dispersion.

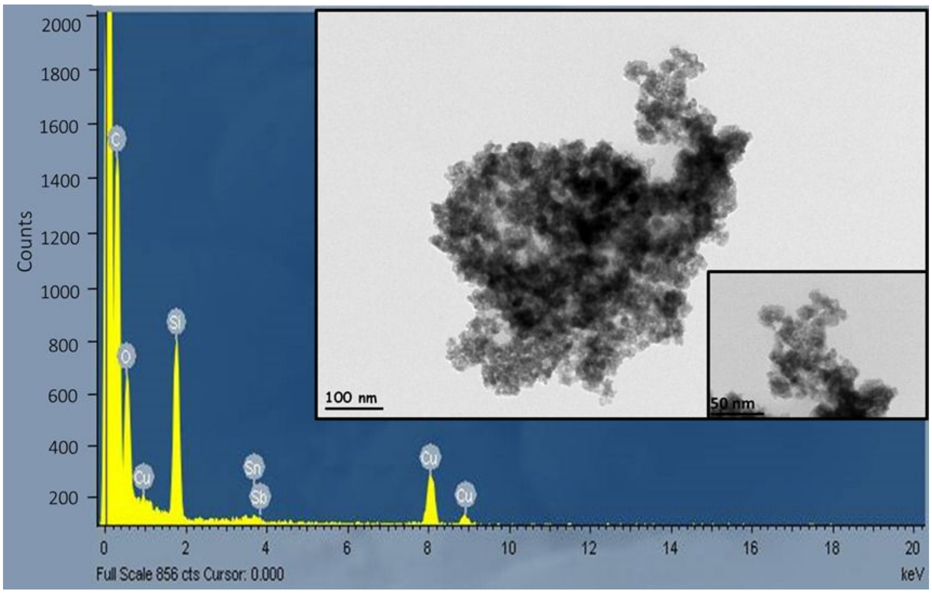

Fig. S2. The EDX results of ATO NPs. Inset. HRTEM images of ATO NPs (scale bars:100 and 50 nm). Sb and Sn exhibit quantitative values in formations with both amorphous and crystalline structures.

References

1. Du, J.; Zhao, R.; Xie, Y.; Li, J., Size-controlled synthesis of SnO2 quantum dots and their gas-sensing performance. *Applied Surface Science* **2015,** *346*, 256-262.
2. Harel, Y.; Lellouche, J.-P., Dual/multiphase MWCNT–antimony-doped tin oxide (ATO) nanoparticle composites: an effective covalent fabrication approach. *Journal of nanoparticle research* **2014,** *16* (8), 2540.
3. Jayachandiran, J.; Yesuraj, J.; Arivanandhan, M.; Muthuraaman, B.; Jayavel, R.; Nedumaran, D., Bifunctional investigation of ultra-small SnO 2 nanoparticle decorated rGO for ozone sensing and supercapacitor applications. *RSC advances* **2021,** *11* (2), 856-866.
4. Subramaniyan, P.; Chen, T.-W.; Chen, S.-M.; Al-Mohaimeed, A. M.; El-Tohamy, M. F.; Elshikh, M. S., In-situ synthesis of novel multifunctionally linked tin oxide on holy reduced graphene oxide: An electrocatalyst for antipsychotic drug sensing. *Materials Research Bulletin* **2023,** *167*, 112443.
5. Li, N.; Meng, Q.; Zhang, N., Dispersion stabilization of antimony-doped tin oxide (ATO) nanoparticles used for energy-efficient glass coating. *Particuology* **2014,** *17*, 49-53.
6. Tan, Q.; Yu, G.; Liao, Y.; Hu, B.; Zhang, X., Preparation of stable aqueous suspensions of antimony-doped tin oxide nanoparticles used for transparent and thermal insulation fluorocarbon coating. *Colloid and Polymer Science* **2014,** *292* (12), 3233-3241.

AutoDock 4.2 atomic parameters (in AD4_parameters.dat)

ADL: Parameters for docking with metal ions in receptor
Dimitry A. Suplatov (genesup@gmail.com)
Thu Mar 19 14:50:59 PDT 2009
Previous message: ADL: correlation between calculated and experimental values.
Next message: ADL: correlation between calculated and experimental values.
Messages sorted by: [date] [thread] [subject] [author]

FE_coeff_vdW 0.1560
FE_coeff_hbond 0.0974
FE_coeff_estat 0.1465
FE_coeff_desolv 0.1159
FE_coeff_tors 0.2744

Notes:
# - To obtain Rij for non H-bonding atoms: arithmetic mean of the two Rii values.
# - To obtain epsij for non H-bonding atoms: geometric mean of the two epsii values.
# - Rij_hb is non-zero for heteroatoms only (zero for H). For an H-bond, look up the heteroatom only.
# - Similarly for epsij_hb. Example: OA–HD H-bonds have Rij_hb = 1.9 Å and epsij_hb = 5.0 kcal/mol.

Columns:
Rii epsii solpar Rij_hb epsij_hb rec_index map_index Type vol hbond bond_index
Format in file: atom_par <Atom> <Rii> <epsii> <solpar> <Rij_hb> <epsij_hb> <rec_index> <map_index> <Type> <vol> <hbond> <bond_index>

atom_par C 4.00 0.150 33.5103 -0.00143 0.0 0.0 0 -1 -1 0 # Non H-bonding
atom_par A 4.00 0.150 33.5103 -0.00052 0.0 0.0 0 -1 -1 0 # Non H-bonding

```
atom_par N   3.50  0.160  22.4493  -0.00162  0.0  0.0  0  -1  -1  1  # Non H-bonding
atom_par NA  3.50  0.160  22.4493  -0.00162  1.9  5.0  4  -1  -1  1  # Acceptor 1 H-bond
atom_par NS  3.50  0.160  22.4493  -0.00162  1.9  5.0  3  -1  -1  1  # Acceptor S Spherical
atom_par OA  3.20  0.200  17.1573  -0.00251  1.9  5.0  3  -1  -1  2  # Acceptor 2 H-bonds
atom_par OS  3.20  0.200  17.1573  -0.00251  1.9  5.0  3  -1  -1  2  # Acceptor S Spherical
atom_par SA  4.00  0.200  33.5103  -0.00214  2.5  1.0  5  -1  -1  6  # Acceptor 2 H-bonds
atom_par S   4.00  0.200  33.5103  -0.00214  0.0  0.0  0  -1  -1  6  # Non H-bonding
atom_par H   2.00  0.020   0.0000   0.00051  0.0  0.0  0  -1  -1  3  # Non H-bonding
atom_par HD  2.00  0.020   0.0000   0.00051  0.0  0.0  2  -1  -1  3  # Donor 1 H-bond
atom_par HS  2.00  0.020   0.0000   0.00051  0.0  0.0  1  -1  -1  3  # Donor S Spherical
atom_par P   4.20  0.200  38.7924  -0.00110  0.0  0.0  0  -1  -1  5  # Non H-bonding
atom_par Br  4.33  0.389  42.5661  -0.00110  0.0  0.0  0  -1  -1  4  # Non H-bonding
atom_par BR  4.33  0.389  42.5661  -0.00110  0.0  0.0  0  -1  -1  4  # Non H-bonding
atom_par Ca  1.98  0.550   2.7700  -0.00110  0.0  0.0  0  -1  -1  4  # Non H-bonding
atom_par CA  1.98  0.550   2.7700  -0.00110  0.0  0.0  0  -1  -1  4  # Non H-bonding
atom_par Cl  4.09  0.276  35.8235  -0.00110  0.0  0.0  0  -1  -1  4  # Non H-bonding
atom_par CL  4.09  0.276  35.8235  -0.00110  0.0  0.0  0  -1  -1  4  # Non H-bonding
atom_par F   3.09  0.080  15.4480  -0.00110  0.0  0.0  0  -1  -1  4  # Non H-bonding
atom_par Fe  1.30  0.010   1.8400  -0.00110  0.0  0.0  0  -1  -1  4  # Non H-bonding
atom_par FE  1.30  0.010   1.8400  -0.00110  0.0  0.0  0  -1  -1  4  # Non H-bonding
atom_par I   4.72  0.550  55.0585  -0.00110  0.0  0.0  0  -1  -1  4  # Non H-bonding
atom_par Mg  1.30  0.875   1.5600  -0.00110  0.0  0.0  0  -1  -1  4  # Non H-bonding
atom_par MG  1.30  0.875   1.5600  -0.00110  0.0  0.0  0  -1  -1  4  # Non H-bonding
atom_par Mn  1.30  0.875   2.1400  -0.00110  0.0  0.0  0  -1  -1  4  # Non H-bonding
atom_par MN  1.30  0.875   2.1400  -0.00110  0.0  0.0  0  -1  -1  4  # Non H-bonding
atom_par Zn  1.48  0.550   1.7000  -0.00110  0.0  0.0  0  -1  -1  4  # Non H-bonding
atom_par ZN  1.48  0.550   1.7000  -0.00110  0.0  0.0  0  -1  -1  4  # Non H-bonding
atom_par He  2.36  0.056  15.240   -0.00110  0.0  0.0  0  -1  -1  0  # Non H-bonding
atom_par Li  2.45  0.025  12.000   -0.00110  0.0  0.0  0  -1  -1  1  # Non H-bonding
atom_par Be  2.76  0.085  12.000   -0.00110  0.0  0.0  0  -1  -1  1  # Non H-bonding
atom_par B   4.08  0.180  12.052   -0.00110  0.0  0.0  0  -1  -1  0  # Non H-bonding
atom_par Ne  3.24  0.042  15.440   -0.00110  0.0  0.0  0  -1  -1  0  # Non H-bonding
atom_par Na  3.98  0.030  12.000   -0.00110  0.0  0.0  0  -1  -1  1  # Non H-bonding
atom_par Al  4.49  0.505  11.278   -0.00110  0.0  0.0  0  -1  -1  1  # Non H-bonding
atom_par Si  4.30  0.402  12.175   -0.00110  0.0  0.0  0  -1  -1  1  # Non H-bonding
atom_par K   3.81  0.035  12.000   -0.00110  0.0  0.0  0  -1  -1  1  # Non H-bonding
atom_par Sc  3.30  0.019  12.000   -0.00110  0.0  0.0  0  -1  -1  1  # Non H-bonding
atom_par Ti  3.18  0.017  12.000   -0.00110  0.0  0.0  0  -1  -1  1  # Non H-bonding
atom_par V   3.14  0.016  12.000   -0.00110  0.0  0.0  0  -1  -1  1  # Non H-bonding
atom_par Co  2.87  0.014  12.000   -0.00110  0.0  0.0  0  -1  -1  1  # Non H-bonding
atom_par Ni  2.83  0.015  12.000   -0.00110  0.0  0.0  0  -1  -1  1  # Non H-bonding
atom_par Cu  3.50  0.005  12.000   -0.00110  0.0  0.0  0  -1  -1  1  # Non H-bonding
atom_par Ga  4.38  0.415  11.000   -0.00110  0.0  0.0  0  -1  -1  1  # Non H-bonding
atom_par Ge  4.28  0.379  12.000   -0.00110  0.0  0.0  0  -1  -1  1  # Non H-bonding
atom_par As  4.23  0.309  13.000   -0.00110  0.0  0.0  0  -1  -1  1  # Non H-bonding
atom_par Se  4.21  0.291  14.000   -0.00110  0.0  0.0  0  -1  -1  1  # Non H-bonding
atom_par Kr  4.14  0.220  16.000   -0.00110  0.0  0.0  0  -1  -1  1  # Non H-bonding
atom_par Rb  4.11  0.040  12.000   -0.00110  0.0  0.0  0  -1  -1  2  # Non H-bonding
```

```
atom_par Sr  3.64  0.235  12.000   -0.00110  0.0  0.0  0  -1  -1  2  # Non H-bonding
atom_par Y   3.35  0.072  12.000   -0.00110  0.0  0.0  0  -1  -1  1  # Non H-bonding
atom_par Zr  3.12  0.069  12.000   -0.00110  0.0  0.0  0  -1  -1  1  # Non H-bonding
atom_par Nb  3.17  0.059  12.000   -0.00110  0.0  0.0  0  -1  -1  1  # Non H-bonding
atom_par Mo  3.05  0.056  12.000   -0.00110  0.0  0.0  0  -1  -1  1  # Non H-bonding
atom_par Tc  3.00  0.048  12.000   -0.00110  0.0  0.0  0  -1  -1  1  # Non H-bonding
atom_par Ru  2.96  0.056  12.000   -0.00110  0.0  0.0  0  -1  -1  1  # Non H-bonding
atom_par Rh  2.93  0.053  12.000   -0.00110  0.0  0.0  0  -1  -1  1  # Non H-bonding
atom_par Pd  1.34  0.048  12.000   -0.00110  0.0  0.0  0  -1  -1  1  # Non H-bonding
atom_par Ag  3.15  0.036  12.000   -0.00110  0.0  0.0  0  -1  -1  1  # Non H-bonding
atom_par Cd  2.85  0.228  12.000   -0.00110  0.0  0.0  0  -1  -1  1  # Non H-bonding
atom_par In  4.46  0.599  11.000   -0.00110  0.0  0.0  0  -1  -1  1  # Non H-bonding
atom_par Sn  4.39  0.567  12.000   -0.00110  0.0  0.0  0  -1  -1  1  # Non H-bonding
atom_par Sb  4.42  0.449  13.000   -0.00110  0.0  0.0  0  -1  -1  1  # Non H-bonding
atom_par Te  4.47  0.398  14.000   -0.00110  0.0  0.0  0  -1  -1  1  # Non H-bonding
atom_par Xe  4.40  0.332  12.000   -0.00110  0.0  0.0  0  -1  -1  1  # Non H-bonding
atom_par Cs  4.52  0.045  12.000   -0.00110  0.0  0.0  0  -1  -1  2  # Non H-bonding
atom_par Ba  3.70  0.364  12.000   -0.00110  0.0  0.0  0  -1  -1  2  # Non H-bonding
atom_par La  3.52  0.017  12.000   -0.00110  0.0  0.0  0  -1  -1  1  # Non H-bonding
atom_par Ce  3.56  0.013  12.000   -0.00110  0.0  0.0  0  -1  -1  1  # Non H-bonding
atom_par Pr  3.61  0.010  12.000   -0.00110  0.0  0.0  0  -1  -1  1  # Non H-bonding
atom_par Nd  3.58  0.010  12.000   -0.00110  0.0  0.0  0  -1  -1  1  # Non H-bonding
atom_par Pm  3.55  0.009  12.000   -0.00110  0.0  0.0  0  -1  -1  1  # Non H-bonding
atom_par Sm  3.52  0.008  12.000   -0.00110  0.0  0.0  0  -1  -1  1  # Non H-bonding
atom_par Eu  3.49  0.008  12.000   -0.00110  0.0  0.0  0  -1  -1  1  # Non H-bonding
atom_par Gd  3.37  0.009  12.000   -0.00110  0.0  0.0  0  -1  -1  1  # Non H-bonding
atom_par Tb  3.45  0.007  12.000   -0.00110  0.0  0.0  0  -1  -1  1  # Non H-bonding
atom_par Dy  3.43  0.007  12.000   -0.00110  0.0  0.0  0  -1  -1  1  # Non H-bonding
atom_par Ho  3.41  0.007  12.000   -0.00110  0.0  0.0  0  -1  -1  1  # Non H-bonding
atom_par Er  3.39  0.007  12.000   -0.00110  0.0  0.0  0  -1  -1  1  # Non H-bonding
atom_par Tm  3.37  0.006  12.000   -0.00110  0.0  0.0  0  -1  -1  1  # Non H-bonding
atom_par Yb  3.36  0.228  12.000   -0.00110  0.0  0.0  0  -1  -1  1  # Non H-bonding
atom_par Lu  3.64  0.041  12.000   -0.00110  0.0  0.0  0  -1  -1  1  # Non H-bonding
atom_par Hf  3.41  0.072  12.000   -0.00110  0.0  0.0  0  -1  -1  1  # Non H-bonding
atom_par Ta  3.71  0.081  12.000   -0.00110  0.0  0.0  0  -1  -1  1  # Non H-bonding
atom_par W   3.07  0.067  12.000   -0.00110  0.0  0.0  0  -1  -1  1  # Non H-bonding
atom_par Re  2.95  0.066  12.000   -0.00110  0.0  0.0  0  -1  -1  1  # Non H-bonding
atom_par Os  3.12  0.120  12.000   -0.00110  0.0  0.0  0  -1  -1  1  # Non H-bonding
atom_par Ir  2.84  0.073  12.000   -0.00110  0.0  0.0  0  -1  -1  1  # Non H-bonding
atom_par Pt  2.75  0.080  12.000   -0.00110  0.0  0.0  0  -1  -1  1  # Non H-bonding
atom_par Au  3.29  0.039  12.000   -0.00110  0.0  0.0  0  -1  -1  1  # Non H-bonding
atom_par Hg  2.71  0.385  12.000   -0.00110  0.0  0.0  0  -1  -1  1  # Non H-bonding
atom_par Tl  4.35  0.680  11.000   -0.00110  0.0  0.0  0  -1  -1  1  # Non H-bonding
atom_par Pb  4.30  0.663  12.000   -0.00110  0.0  0.0  0  -1  -1  1  # Non H-bonding
atom_par Bi  4.37  0.518  13.000   -0.00110  0.0  0.0  0  -1  -1  1  # Non H-bonding
atom_par Po  4.71  0.325  14.000   -0.00110  0.0  0.0  0  -1  -1  1  # Non H-bonding
atom_par At  4.75  0.284  15.000   -0.00110  0.0  0.0  0  -1  -1  1  # Non H-bonding
atom_par Rn  4.77  0.248  16.000   -0.00110  0.0  0.0  0  -1  -1  1  # Non H-bonding
```

atom_par Fr 4.90 0.050 12.000   -0.00110 0.0 0.0 0 -1 -1 2 # Non H-bonding
atom_par Ra 3.68 0.404 12.000   -0.00110 0.0 0.0 0 -1 -1 2 # Non H-bonding
atom_par Ac 3.48 0.033 12.000   -0.00110 0.0 0.0 0 -1 -1 1 # Non H-bonding
atom_par Th 3.40 0.026 12.000   -0.00110 0.0 0.0 0 -1 -1 1 # Non H-bonding
atom_par Pa 3.42 0.022 12.000   -0.00110 0.0 0.0 0 -1 -1 1 # Non H-bonding
atom_par U   3.40 0.022 12.000   -0.00110 0.0 0.0 0 -1 -1 1 # Non H-bonding
atom_par Np 3.42 0.019 12.000   -0.00110 0.0 0.0 0 -1 -1 1 # Non H-bonding
atom_par Pu 3.42 0.016 12.000   -0.00110 0.0 0.0 0 -1 -1 1 # Non H-bonding
atom_par Am 3.38 0.014 12.000   -0.00110 0.0 0.0 0 -1 -1 1 # Non H-bonding
atom_par Cm 3.33 0.014 12.000   -0.00110 0.0 0.0 0 -1 -1 1 # Non H-bonding
atom_par Bk 3.34 0.013 12.000   -0.00110 0.0 0.0 0 -1 -1 1 # Non H-bonding
atom_par Cf 3.31 0.013 12.000   -0.00110 0.0 0.0 0 -1 -1 1 # Non H-bonding
atom_par E   3.30 0.012 12.000   -0.00110 0.0 0.0 0 -1 -1 1 # Non H-bonding
atom_par Fm 3.29 0.012 12.000   -0.00110 0.0 0.0 0 -1 -1 1 # Non H-bonding

Docking Parameter File (.dpf) for tubulin heterodimer

Setting	Comment
**parameter_file AD4_parameters.dat**	
autodock_parameter_version 4.2	used by autodock to validate parameter set
outlev 1	diagnostic output level
intelec	calculate internal electrostatics
seed pid time	seeds for random generator
ligand_types Sb Sn OA	atoms types in ligand
fld tubulin_heterodimer.maps.fld	grid_data_file
map tubulin_heterodimer.Sb.map	atom-specific affinity map
map tubulin_heterodimer.Sn.map	atom-specific affinity map
map tubulin_heterodimer.OA.map	atom-specific affinity map
elecmap tubulin_heterodimer.e.map	electrostatics map
desolvmap tubulin_heterodimer.d.map	desolvation map
move SnO2.pdbqt	small molecule
about -23.347 126.607 17.136	small molecule center
tran0 random	initial coordinates/A or random
quaternion0 random	initial orientation
dihe0 random	initial dihedrals (relative) or random
torsdof 0	torsional degrees of freedom
rmstol 2.0	cluster_tolerance/A
extnrg 1000.0	external grid energy
e0max 0.0 10000	max initial energy; max number of retries
ga_pop_size 150	number of individuals in population
ga_num_evals 5000000	maximum number of energy evaluations
ga_num_generations 27000	maximum number of generations
ga_elitism 1	number of top individuals to survive to next generation
ga_mutation_rate 0.02	rate of gene mutation
ga_crossover_rate 0.8	rate of crossover
ga_window_size 10	

Setting	Comment
ga_cauchy_alpha 0.0	Alpha parameter of Cauchy distribution
ga_cauchy_beta 1.0	Beta parameter Cauchy distribution
set_ga	set the above parameters for GA or LGA
sw_max_its 300	iterations of Solis & Wets local search
sw_max_succ 4	consecutive successes before changing rho
sw_max_fail 4	consecutive failures before changing rho
sw_rho 1.0	size of local search space to sample
sw_lb_rho 0.01	lower bound on rho
ls_search_freq 0.06	probability of performing local search on individual
set_psw1	set the above pseudo-Solis & Wets parameters
unbound_model bound	state of unbound ligand
ga_run 20	do this many hybrid GA-LS runs
analysis	perform a ranked cluster analysis

Docking Parameter File (.dpf) for B-DNA dodecamer

Setting	Comment
**parameter_file AD4_parameters.dat**	
autodock_parameter_version 4.2	used by autodock to validate parameter set
outlev 1	diagnostic output level
intelec	calculate internal electrostatics
seed pid time	seeds for random generator
ligand_types Sb Sn OA	atoms types in ligand
fld 1BNA.maps.fld	grid_data_file
map 1BNA.Sb.map	atom-specific affinity map
map 1BNA.Sn.map	atom-specific affinity map
map 1BNA.OA.map	atom-specific affinity map
elecmap 1BNA.e.map	electrostatics map
desolvmap 1BNA.d.map	desolvation map
move SnO2.pdbqt	small molecule
about 4.368 4.368 2.405	small molecule center
tran0 random	initial coordinates/A or random
quaternion0 random	initial orientation
dihe0 random	initial dihedrals (relative) or random
torsdof 0	torsional degrees of freedom
rmstol 2.0	cluster_tolerance/A
extnrg 1000.0	external grid energy
e0max 0.0 10000	max initial energy; max number of retries
ga_pop_size 150	number of individuals in population
ga_num_evals 5000000	maximum number of energy evaluations
ga_num_generations 27000	maximum number of generations
ga_elitism 1	number of top individuals to survive to next generation
ga_mutation_rate 0.02	rate of gene mutation
ga_crossover_rate 0.8	rate of crossover

Setting	Comment
ga_window_size 10	
ga_cauchy_alpha 0.0	Alpha parameter of Cauchy distribution
ga_cauchy_beta 1.0	Beta parameter Cauchy distribution
set_ga	set the above parameters for GA or LGA
sw_max_its 300	iterations of Solis & Wets local search
sw_max_succ 4	consecutive successes before changing rho
sw_max_fail 4	consecutive failures before changing rho
sw_rho 1.0	size of local search space to sample
sw_lb_rho 0.01	lower bound on rho
ls_search_freq 0.06	probability of performing local search on individual
set_psw1	set the above pseudo-Solis & Wets parameters
unbound_model bound	state of unbound ligand
ga_run 20	do this many hybrid GA-LS runs
analysis	perform a ranked cluster analysis

Grid Parameter File (.gpf) for tubulin heterodimer

Setting	Comment
**parameter_file AD4_parameters.dat**	
npts 60 60 60	num.grid points in xyz
gridfld tubulin_heterodimer.maps.fld	grid_data_file
spacing 0.375	spacing(A)
receptor_types A C HD N NA OA SA	receptor atom types
ligand_types Sb Sn OA	ligand atom types
receptor tubulin_heterodimer.pdbqt	macromolecule
gridcenter 16.228 79.399 42.581	xyz-coordinates or auto
smooth 0.5	store minimum energy w/in rad(A)
map tubulin_heterodimer.Sb.map	atom-specific affinity map
map tubulin_heterodimer.Sn.map	atom-specific affinity map
map tubulin_heterodimer.OA.map	atom-specific affinity map
elecmap tubulin_heterodimer.e.map	electrostatic potential map
dsolvmap tubulin_heterodimer.d.map	desolvation potential map
dielectric -0.1465	<0, AD4 distance-dep.diel;>0, constant

Grid Parameter File (.gpf) for B-DNA dodecamer

Setting	Comment
**parameter_file AD4_parameters.dat**	
npts 60 60 120	num.grid points in xyz
gridfld 1BNA.maps.fld	grid_data_file
spacing 0.375	spacing(A)
receptor_types A C HD N OA P	receptor atom types
ligand_types Sb Sn OA	ligand atom types
receptor 1BNA.pdbqt	macromolecule

**Setting**	**Comment**
gridcenter 14.779 20.976 8.804	xyz-coordinates or auto
smooth 0.5	store minimum energy w/in rad(A)
map 1BNA.Sb.map	atom-specific affinity map
map 1BNA.Sn.map	atom-specific affinity map
map 1BNA.OA.map	atom-specific affinity map
elecmap 1BNA.e.map	electrostatic potential map
dsolvmap 1BNA.d.map	desolvation potential map
dielectric -0.1465	<0, AD4 distance-dep.diel;>0, constant
